# Interactions between gastric microbiota and metabolites in gastric cancer

**DOI:** 10.1038/s41419-021-04396-y

**Published:** 2021-11-24

**Authors:** Daofeng Dai, Yan Yang, Jieqing Yu, Tianfeng Dang, Wenjing Qin, Lisong Teng, Jing Ye, Hongqun Jiang

**Affiliations:** 1 Jiangxi Otorhinolaryngology Head and Neck Surgery Institute, Department of Otorhinolaryngology Head and Neck Surgery, The First Affiliated Hospital of Nanchang University, Nanchang, Jiangxi China; 2 Department of Surgical Oncology, The First Affiliated Hospital, School of Medicine, Zhejiang University, Hangzhou, Zhejiang, China; 3 Human Genetic Resources Center, The First Affiliated Hospital of Nanchang University, Nanchang, Jiangxi China

**Keywords:** Cancer metabolism, Gastrointestinal cancer

## Abstract

The development and progression of gastric cancer (GC) is greatly influenced by gastric microbiota and their metabolites. Here, we characterized the gastric microbiome and metabolome profiles of 37 GC tumor tissues and matched non-tumor tissues using 16s rRNA gene sequencing and ultrahigh performance liquid chromatography tandem mass spectrometry, respectively. Microbial diversity and richness were higher in GC tumor tissues than in non-tumor tissues. The abundance of *Helicobacter* was increased in non-tumor tissues, while the abundance of *Lactobacillus*, *Streptococcus*, *Bacteroides*, *Prevotella*, and 6 additional genera was increased in the tumor tissues. The untargeted metabolome analysis revealed 150 discriminative metabolites, among which the relative abundance of the amino acids, carbohydrates and carbohydrate conjugates, glycerophospholipids, and nucleosides was higher in tumor tissues compared to non-tumor tissues. The targeted metabolome analysis further demonstrated that the combination of 1-methylnicotinamide and *N*-acetyl-D-glucosamine-6-phosphate could serve as a robust biomarker for distinction between GC tumors and non-tumor tissues. Correlation analysis revealed that *Helicobacter* and *Lactobacillus* were negatively and positively correlated with the majority of differential metabolites in the classes of amino acids, carbohydrates, nucleosides, nucleotides, and glycerophospholipids, respectively, suggesting that *Helicobacter* and *Lactobacillus* might play a role in degradation and synthesis of the majority of differential metabolites in these classes, respectively. *Acinetobacter*, *Comamonas*, *Faecalibacterium*, *Sphingomonas*, and *Streptococcus* were also significantly correlated with many differential amino acids, carbohydrates, nucleosides, nucleotides, and glycerophospholipids. In conclusion, the differences in metabolome profiles between GC tumor and matched non-tumor tissues may be partly due to the collective activities of *Helicobacter*, *Lactobacillus*, and other bacteria, which eventually affects GC carcinogenesis and progression.

## Introduction

Gastric cancer (GC) is a prominent malignant tumor worldwide, particularly in Asia [1]. According to the latest cancer statistics in China, GC was responsible for over 679,100 new cases and 498,000 deaths in 2015, making it the second most frequently diagnosed cancer as well as the second leading cause of deaths related with cancer [2]. Infection with *Helicobacter pylori* is widely regarded as a high-risk factor for the development of GC as the majority of GC cases can be related to *H. pylori* [3]. Other risk factors include smoking, gender, and the consumption of smoked and high-salt foods [4].

Due to the acidic environment of the human stomach, it was previously believed that the stomach was not suitable for the growth of other microorganisms and was exclusively colonized by *H. pylori*. However, advances in sequencing technology have proven that the stomach is inhabited by a robust microbiota [5]. Previous studies have found that the microbial diversity in patients with intestinal metaplasia and GC were significantly decreased compared to in patients with superficial gastritis [6, 7]. Moreover, other studies showed that GC was associated with increased microbial diversity and richness [8, 9]. A study using high-throughput sequencing techniques on a cohort of 276 Chinese patients with GC found that bacterial diversity and richness were lower in peritumoral and tumoral tissues than in non-tumor tissues, and that the composition of gastric microbiota was significantly altered in the different stomach microhabitats [10].

The development of tumors is affected not only by microbiota but also by their metabolites [11]. Kaji et al. used capillary electrophoresis time-of-flight mass spectrometry to quantify 93 metabolites in cancer and adjacent non-cancerous tissues from 140 patients with GC, revealing that β-alanine was both a significant predictor of peritoneal recurrence and a prognostic factor for GC [12]. Additionally, other studies have investigated the metabolite differences between non-tumor and cancerous tissues from patients with GC [13–15]. However, the number of metabolites quantified in these studies were very small, resulting in a lack of information on a large number of metabolites. Thus, further studies analyzing more metabolites are urgently needed.

Human metabolites are a mixture of products from human genome and bacterial genome, which may be more affected by human genome, but bacterial genome also plays a role in the biosynthesis and degradation of human metabolites. Erawijantari et al. evaluated the influence of gastrectomy as a GC treatment on fecal microbiome and metabolome profiles [16]. Nevertheless, the contributions of microbes to metabolite production and degradation in GC tissues remains unclear.

In this study, we performed 16s rRNA gene sequencing on tumor tissues and matched non-tumor tissues from 37 patients with GC to characterize the gastric microbiota. We also performed untargeted metabolome analysis of the 37 paired GC tissue samples using ultrahigh performance liquid chromatography tandem mass spectrometry (UHPLC-MS/MS) to characterize the gastric metabolome profiles, and combined this analysis with the GC tissue microbiome profiles.

## Results

### Altered gastric microbiota in GC tumor tissues compared with matched non-tumor tissues

**Fig. 1:**
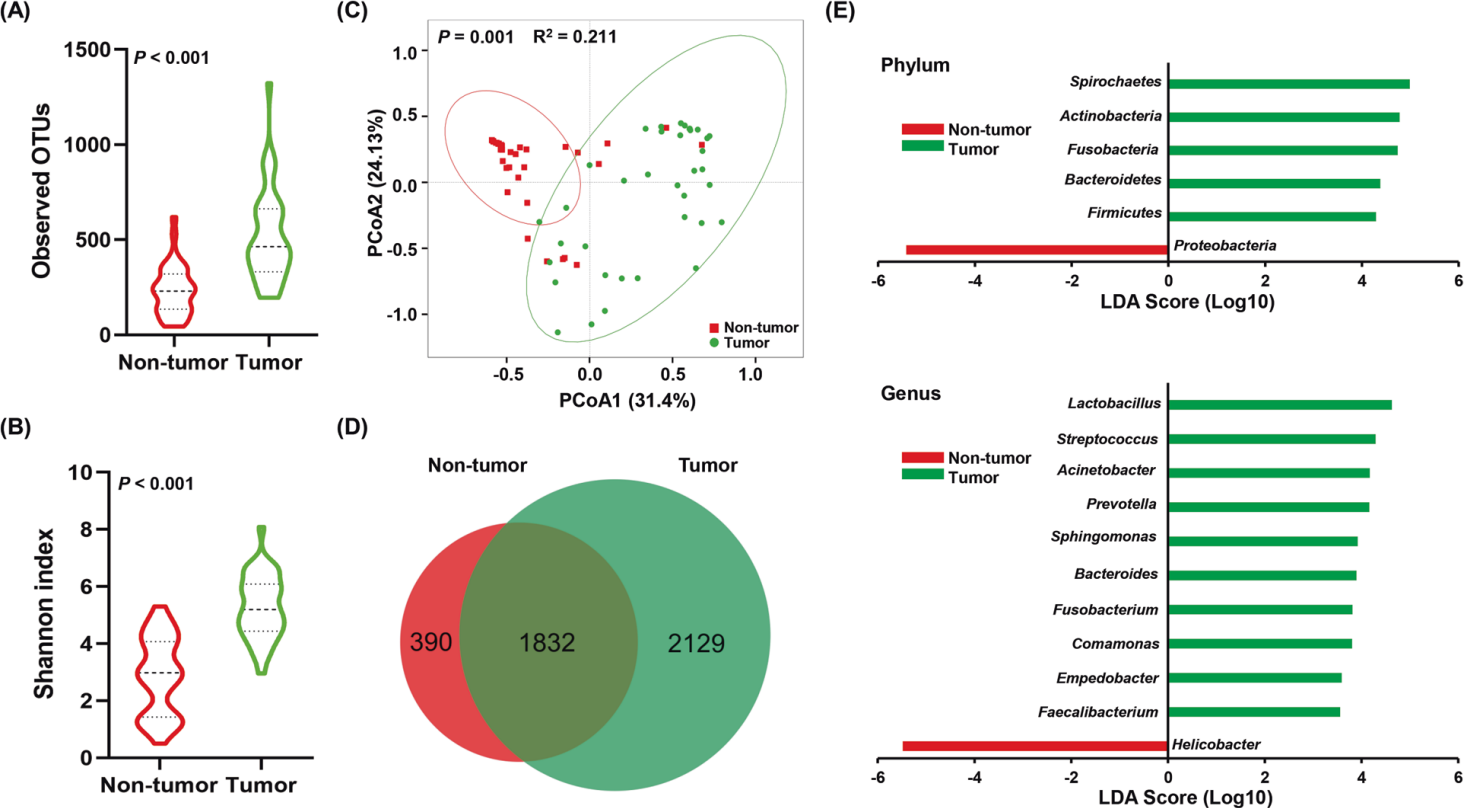
Altered gastric microbiota in 37 gastric cancer (GC) tissues compared with matched non-tumor tissues. **A, B** The observed OTUs and Shannon indices were used to evaluate the microbial diversity of the paired tumor and non-tumor tissues. The Wilcoxon matched-pairs signed rank test was performed. **C** Principal coordinate analysis (PCoA) of the weighted UniFrac distance demonstrated that the non-tumor and tumor tissues showed two distinct clusters. **D** The Venn diagram illustrates the overlapped OTUs between the paired GC tumor tissues and non-tumor tissues. **E** Differential taxa at the phylum and genus levels identified by linear discriminant analysis (LDA) effect size (LEfSe) analysis (LDA > 3.5, *Q* < 0.05).

As shown in Table S1, the 16s rRNA gene sequencing produced a median of 80,110 clean reads for 37 paired tumor and non-tumor tissues (Cohort 1, Table 1). To measure differences in microbial diversity between the groups, alpha diversity was analyzed. The observed OTUs, which reflects the species richness, was significantly higher in tumor tissues than in non-tumor tissue (464.00 vs. 231.00; *P* < 0.001; Fig. 1A). The Shannon index, which measures species richness and evenness, was also significantly higher in tumor tissues than in non-tumor tissue (5.20 vs. 2.98; *P* < 0.001; Fig. 1B). To compare the composition of the microbial community between the non-tumor and tumor tissues, we analyzed beta diversity. The weighted UniFrac principal coordinate analysis (PCoA) showed that significant clustering was detected between groups (PERMANOVA, *R*^2^ = 0.211, *P* = 0.001, Fig. 1C). As shown in the Venn diagram, 2222 and 3961 OTUs were detected in the non-tumor and tumor tissues, respectively, with 1832 OTUs concurrent in the two groups (Fig. 1D). To identify specific microbial communities associated with GC, we analyzed the composition of the gastric microbiota in non-tumor and tumor tissues using LEfSe analysis. A total of 64 discriminative taxa at all taxonomic levels from phylum to genus were identified (LDA > 3.5, *Q* < 0.05). At the phylum level, the abundance of *Proteobacteria* was increased in the non-tumor tissues, whereas the abundance of *Firmicutes*, *Bacteroidetes*, *Actinobacteria*, *Fusobacteria*, and *Spirochetes* was enriched in the tumor tissues (Fig. 1E). At the genus level, the abundance of *Helicobacter* was elevated in the non-tumor tissues, whereas the abundance of *Lactobacillus*, *Streptococcus*, *Acinetobacter*, *Prevotella*, *Sphingomonas*, *Bacteroides*, *Fusobacterium*, *Comamonas*, *Empedobacter*, and *Faecalibacterium* was increased in the tumor tissues (Fig. 1E).

**Table 1:** Clinicopathological characteristics of patients with GC in this study.

Characteristics	Cohort 1	Cohort 2
Total number	37	20
Gender (no.)		
Female	11	6
Male	26	14
Age (years, mean ± SD)	66.30 ± 10.44	62.20 ± 13.74
Weight (kg, mean ± SD)	60.72 ± 11.82	56.10 ± 9.23
Height (cm, mean ± SD)	165.05 ± 8.46	161.40 ± 8.13
BMI (mean ± SD)	22.13 ± 3.04	21.49 ± 2.77
Complications (no.)		
Hypertension	8	0
Diabetes mellitus	2	0
Tumor localization (no.)		
Proximal stomach	15	4
Antrum	14	8
Body/fundus	8	8
Tumor differentiation (no.)		
Moderately differentiated	2	5
Moderately–poorly differentiated	13	6
Poorly differentiated	22	7
Unknown	0	2
Lauren typing (no.)		
Intestinal type	13	10
Diffuse type	11	5
Mixed type	13	5
Tumor stage (no.)		
I	4	1
II	12	3
III	21	16

### Differences in the metabolome profiles between GC tumor and non-tumor tissues

**Fig. 2:**
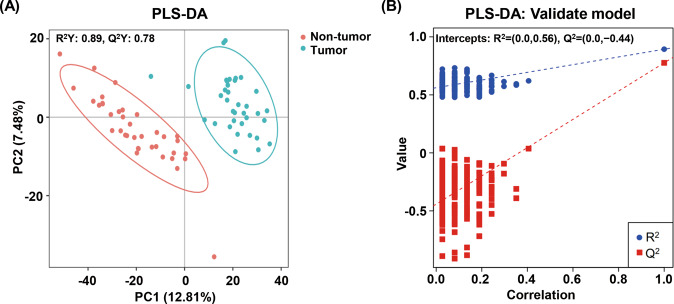
The metabolome profiles of gastric cancer tissues were different from those of matched non-tumor tissues. **A** PLS-DA showed that tumor tissues and non-tumor tissues were separated into two distinct clusters. **B** The test for PLS-DA model showed that the PLS-DA model for this study was valid. PLS-DA, partial least-squares discriminant analysis. QC, quality control. The QC samples were obtained by mixing the equal amounts of metabolites extracted from all samples, which were used for evaluation of the stability of the instrument.

**Fig. 3:**
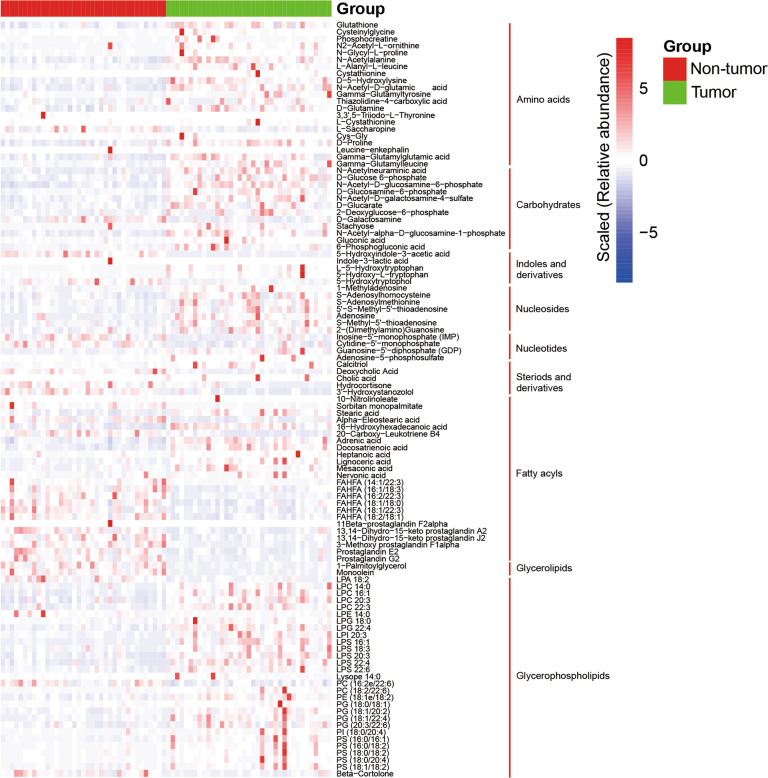
The heat map shows the differential metabolites between the paired gastric cancer tissues and non-tumor tissues. The heat map shows the scaled relative abundance (Lg) of 109 differential metabolites (VIP > 1 and *Q* value < 0.05 and fold change (FC) ≥ 2 or FC ≤ 0.5). The discriminative metabolites from top to bottom are amino acids, carbohydrates and carbohydrate conjugates, indoles and derivatives, nucleosides, nucleotides, steroids and derivatives, fatty acyls, glycerolipids, and glycerophospholipids. The differential metabolites were classified using the Human Metabolome Database. *Q* value, corrected *P* value.

Since the diversity and composition of the gastric microbiota were different between the non-tumor and tumor tissues from GC patients, we hypothesized that changes in the metabolomic pathways may be partially influenced by gastric microbiota in GC patients. Thus, untargeted metabolome analysis of the tissue samples (37 paired GC tissue samples) was performed using UHPLC-MS/MS, and 1198 metabolites were quantified in the positive and negative modes. The PLS-DA score plot showed that the tumor and non-tumor tissues were separated into two distinct clusters (*R*^2^Y = 0.89 and *Q*^2^Y = 0.78) (Fig. 2A). The test for the PLS-DA model showed that the *R*^2^ value was larger than the *Q*^2^ value, and that the *Q*^2^ regression line had a negative intercept (*R*^2^ = [0.0, 0.56], *Q*^2^ = [0.0, −0.44]), indicating that the PLS-DA model for this study was valid (Fig. 2B). We observed 150 metabolites with significantly differential relative abundance between the non-tumor and tumor tissues (variable importance in projection (VIP) > 1 and *Q* value < 0.05 and FC ≥ 2 or FC ≤ 0.5) (Table S2), which included 21 amino acids, 12 carbohydrates and carbohydrate conjugates, 24 fatty acyls, 29 glycerophospholipids, 5 indoles and derivatives, 7 nucleosides, 4 nucleotides, 5 steroids and derivatives, 3 benzenoids, and 2 glycerolipids (Fig. 3). The relative abundance of these metabolites in the classes of amino acids, carbohydrates and carbohydrate conjugates, glycerophospholipids, and nucleosides was higher in the tumor tissues than in the non-tumor tissues (Fig. 3). As for the metabolites of the fatty acyl class, the relative abundance of fatty acid esters of hydroxy fatty acids and prostaglandins was decreased in the tumor tissues compared to the non-tumor tissues. The majority of the remaining metabolites in this class exhibited increased relative abundance in the tumor tissues compared with the non-tumor tissues.

### Identification of metabolite biomarkers for discriminating tumor from non-tumor tissues

**Fig. 4:**
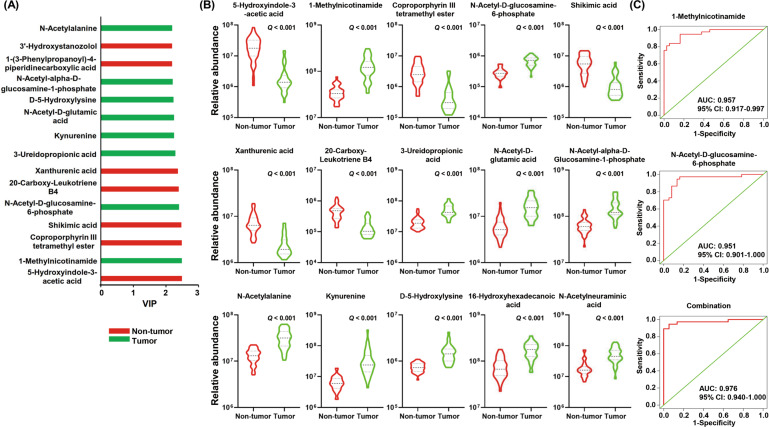
Identification of metabolite biomarkers for discriminating gastric tumor tissues from non-tumor tissues. **A** The top 15 metabolites according to VIP values are displayed. VIP, variable importance in projection. **B** Among the 15 metabolites, the relative abundance of 8 metabolites was higher, but the relative abundance of the rest of the metabolites was lower in the tumor tissues than the non-tumor tissues. *Q* value, corrected *P* value. **C** ROC analysis for 1-methylnicotinamide, *N*-acetyl-D-glucosamine-6-phosphate, and the combination of the two metabolites. ROC, receiver operating curve.

To identify metabolite biomarkers for discriminating between tumor and non-tumor tissues, we selected the top 15 metabolites according to VIP values (Fig. 4A). Among the 15 metabolites, the relative abundance of 8 metabolites was higher in the tumor tissues than in the non-tumor tissues (Fig. 4B). Next, we preformed the receiver operating curve (ROC) analysis to evaluate the diagnostic accuracy of the 8 metabolites in discriminating between tumor and non-tumor tissues. Metabolites with an area under the curve (AUC) < 0.95 were eliminated. Finally, we obtained two candidate biomarkers, 1-methylnicotinamide and *N*-acetyl-D-glucosamine-6-phosphate. Their corresponding AUCs were 0.957 (95% CI: 0.917–0.997) and 0.951 (95% CI: 0.901–1.000), respectively (Fig. 4C). The AUC for the combination of the two metabolites was 0.976 (95% CI: 0.940–1.000) (Fig. 4C). These results suggest that the combination of 1-methylnicotinamide and *N*-acetyl-D-glucosamine-6-phosphate may serve as a potential biomarker for discrimination between GC tumors and non-tumor tissues.

### Validation of metabolite biomarkers for discriminating tumor from non-tumor tissues

**Fig. 5:**
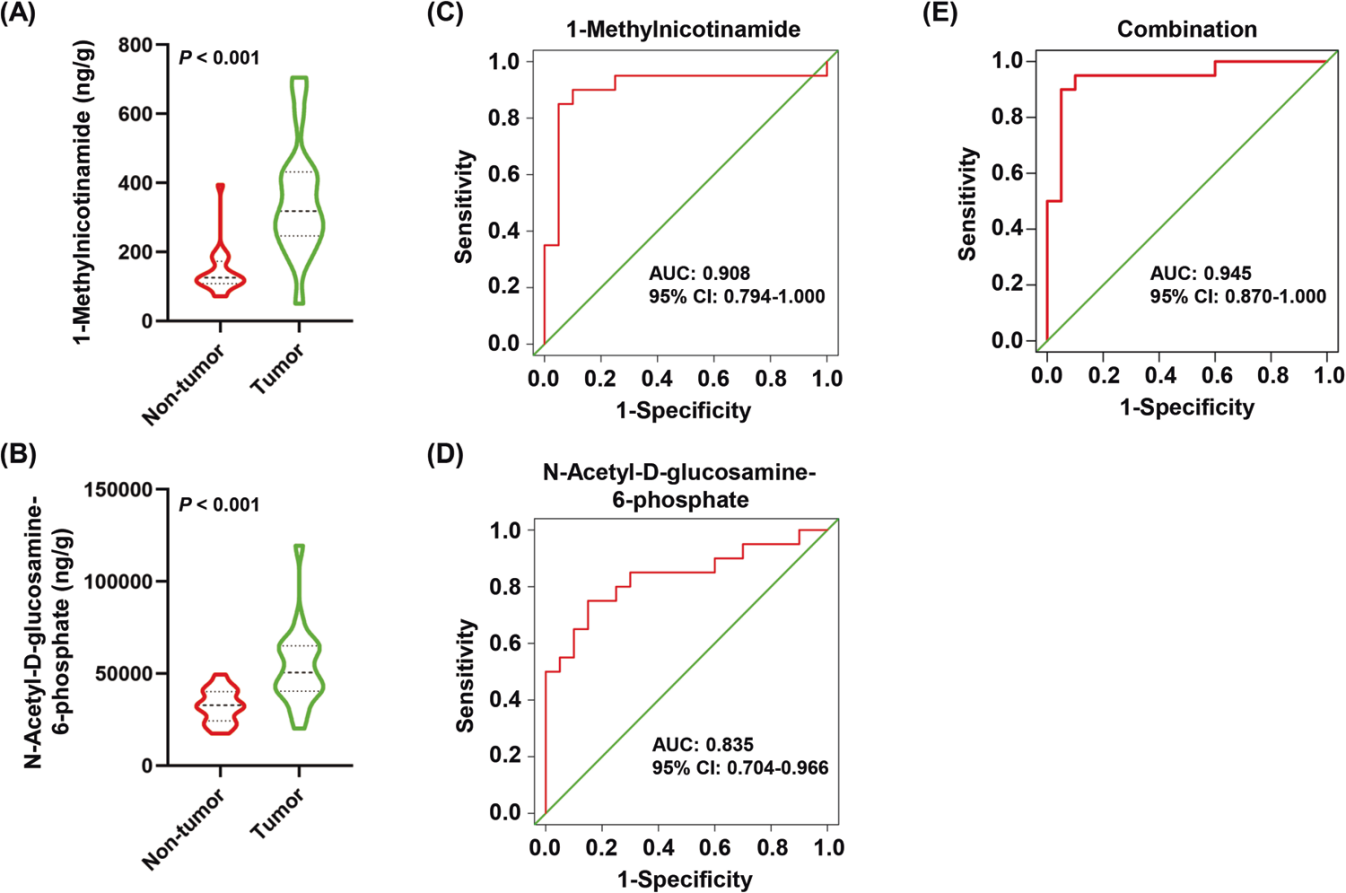
Validation of metabolite biomarkers for distinguishing between gastric tumor and non-tumor tissues. **A** The concentrations of 1-methylnicotinamide was compared between tumor tissues and matched non-tumor tissues from 20 patients with gastric cancer (GC). **B** Comparison of the concentration of *N*-acetyl-D-glucosamine-6-phosphate between 20 paired GC tumor and non-tumor tissues. **C–E** ROC analysis for 1-methylnicotinamide (**C**), *N*-acetyl-D-glucosamine-6-phosphate (**D**), and the combination of the two metabolites (**E**). ROC, receiver operating curve.

To validate the combination of 1-methylnicotinamide and *N*-acetyl-D-glucosamine-6-phosphate as the biomarkers for discrimination between GC tumors and non-tumor tissues, we recruited 20 additional patients with GC (Cohort 2) for targeted metabolomics (Table 1). As shown in Fig. 5A, B, the concentrations of 1-methylnicotinamide and *N*-acetyl-D-glucosamine-6-phosphate were both significantly higher in GC tumor tissues than in non-tumor tissues (*P* < 0.001). The AUCs for 1-methylnicotinamide and *N*-acetyl-D-glucosamine-6-phosphate were 0.908 (95% CI: 0.794–1.000) and 0.835 (95% CI: 0.704–0.966), respectively (Fig. 5C, D). The AUC for the combination of the two metabolites was 0.945 (95% CI: 0.870–1.000) (Fig. 5E). These results show that the combination of 1-methylnicotinamide and *N*-acetyl-D-glucosamine-6-phosphate may serve as a robust biomarker for distinction between GC tumors and non-tumor tissues.

### KEGG enrichment analysis of differential metabolites

**Fig. 6:**
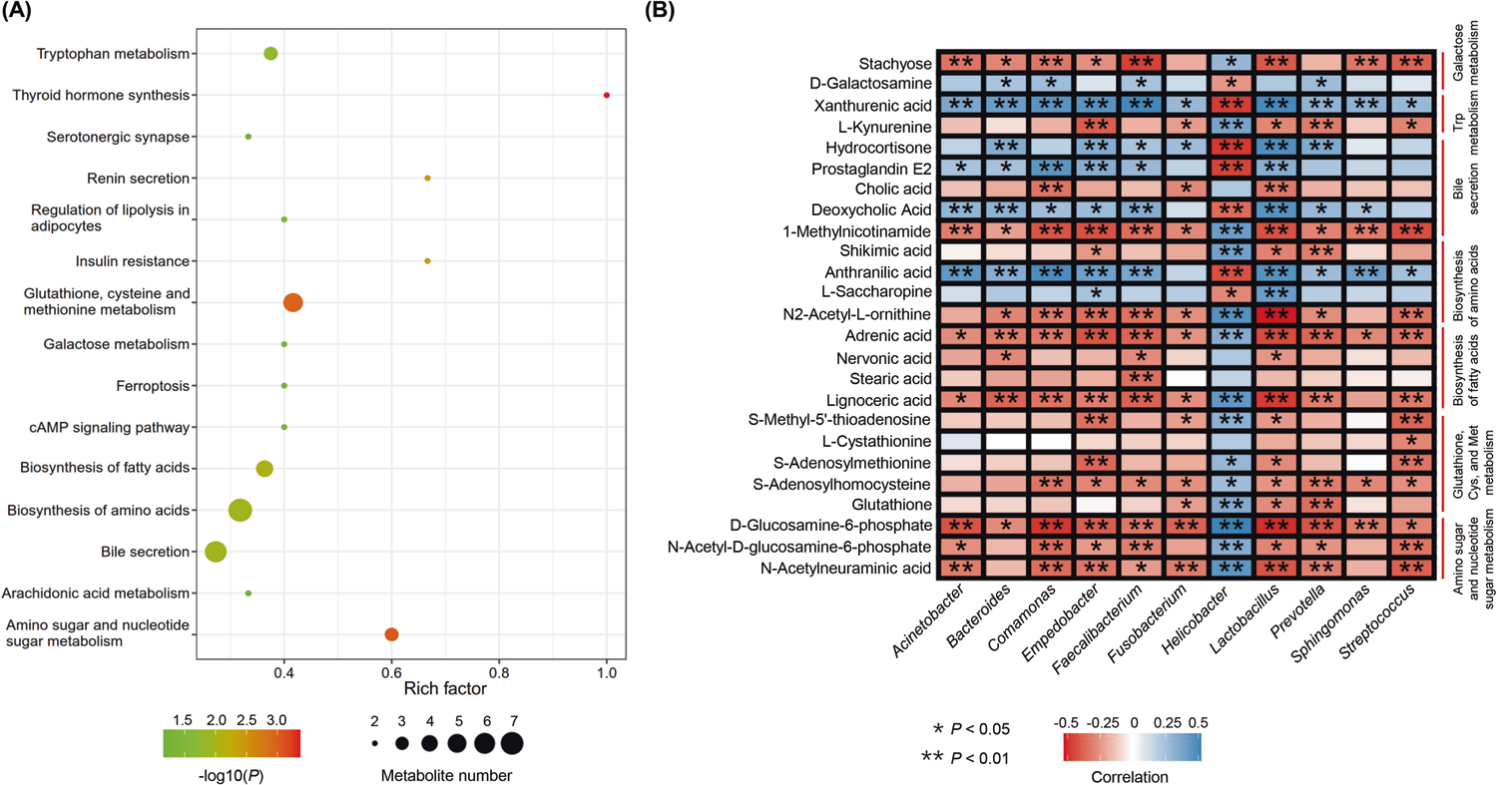
Pathway analysis for metabolites and integrated analysis of microbiota and metabolites. **A** The functions of these metabolites and metabolic pathways were studied using the KEGG database, and enriched pathways were displayed by a bubble plot. **B** The association between 11 discriminative genera and 25 differential metabolites in the main enriched pathways were analyzed using the Spearman’s correlation method. Red, positive correlations; blue, negative correlations. *P value < 0.05; **P value < 0.01.

To determine the main metabolic pathways and signal pathways correlated with the differential metabolites in the non-tumor and tumor tissues, KEGG enrichment analysis was performed. Figure 6A showed 150 discriminative metabolites scattered through multiple pathways, including tryptophan metabolism, amino acid biosynthesis, fatty acid biosynthesis, bile secretion, and galactose metabolism, etc. Furthermore, glutathione, cysteine and methionine metabolism, amino sugar and nucleotide sugar metabolism, and thyroid hormone synthesis pathways were significantly enriched. The pathways of glutathione, cysteine, and methionine metabolism (5 differential metabolites), biosynthesis of amino acids (7 differential metabolites), and bile secretion (6 differential metabolites) contained more differential metabolites than the other pathways.

### The association between discriminative genera and metabolites in different pathways

The Spearman’s correlation analysis was used to assess the association between 11 discriminative genera and 25 differential metabolites in the main enriched pathways, which showed that the differential metabolites were not only correlated with the genus *Helicobacter* but also with other genera (Fig. 6B). *Helicobacter* was enriched in non-tumor tissues and exhibited a significantly negative correlation with the differential metabolites in pathways of amino sugar and nucleotide sugar metabolism; glutathione, cysteine, and methionine metabolism; and biosynthesis of fatty acids. This indicates that *Helicobacter* plays a role in the degradation of these metabolites. All differential metabolites in the pathway of amino sugar and nucleotide sugar metabolism were significantly positively associated with *Lactobacillus*, *Streptococcus*, *Prevotella*, *Acinetobacter*, *Comamonas*, *Empedobacter*, and *Faecalibacterium*. *Lactobacillus* and *Streptococcus* both exhibited significantly positive correlation with four out of five differential metabolites in the pathway of glutathione, cysteine, and methionine metabolism. All the differential metabolites in the biosynthesis pathway of fatty acids were significantly positively correlated with *Faecalibacterium*. Three out of four differential metabolites in this pathway exhibited significantly positive correlation with *Lactobacillus* and *Bacteroides*. These results suggested that *Lactobacillus*, *Streptococcus*, *Prevotella*, *Acinetobacter*, *Comamonas*, *Empedobacter*, *Faecalibacterium*, and *Bacteroides* contribute greatly to the synthesis of the differential metabolites in the respective pathways. *Lactobacillus* significantly correlated with all the differential metabolites in the bile secretion pathway, the biosynthesis pathway of amino acids, and the tryptophan metabolism pathway. *Helicobacter* also showed a significant correlation with several discriminative metabolites in these pathways.

### The relationship between discriminative genera and metabolites in different classes

**Fig. 7:**
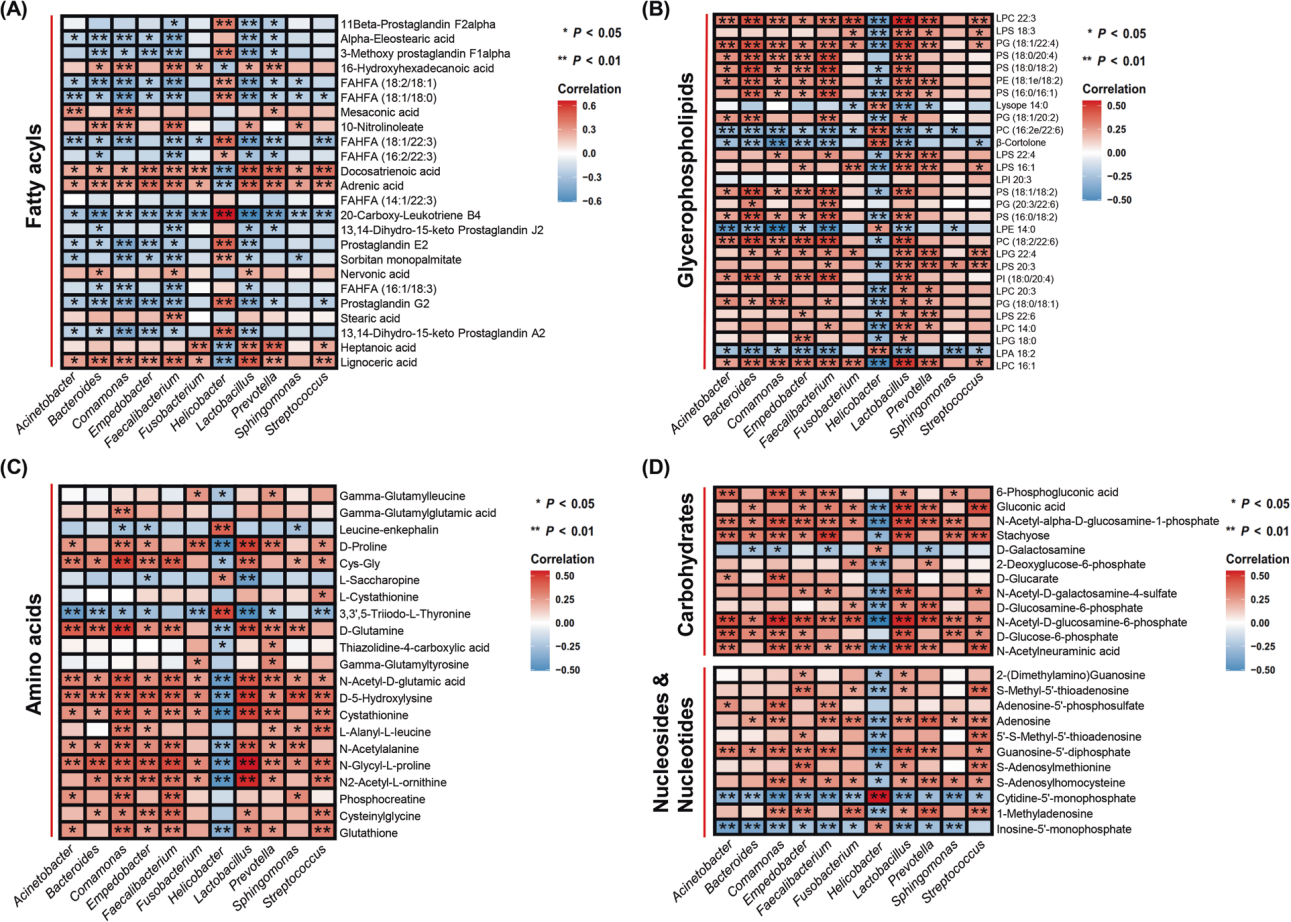
The analysis of correlation between differential genera and metabolites in different classes. **A–D** The analysis of correlation between 11 differential genera and discriminative metabolites in the classes of fatty acyls (*n* = 24) (**A**), differential glycerophospholipids (*n* = 29) (**B**), amino acids (*n* = 21) (**C**), carbohydrates (*n* = 12), nucleosides (*n* = 7), and nucleotides (*n* = 4) (**D**). Red, positive correlations; blue, negative correlations. *P value < 0.05; **P value < 0.01.

The analysis of association between differential genera and metabolites in different classes was performed. As shown in Fig. 7A, *Helicobacter* significantly correlated with 16 fatty acyls, while *Lactobacillus* was significantly associated with 21 fatty acyls, suggesting that the metabolites in this class were strongly influenced by *Helicobacter* and *Lactobacillus*. *Helicobacter* was negatively correlated with the majority of differential metabolites in the classes of amino acids, carbohydrates, nucleosides, nucleotides, and glycerophospholipids; however, *Lactobacillus* was positively associated with the majority of the differential metabolites in these classes (Fig. 7B–D). These results indicated that *Helicobacter* and *Lactobacillus* might contribute to degradation and synthesis of metabolites in these classes, respectively. *Bacteroides* exhibited significant association with 18 fatty acyls and 19 glycerophospholipids, while *Faecalibacterium* showed significant correlation with 21 fatty acyls and 22 glycerophospholipids (Fig. 7A, B). These results suggested that *Bacteroides* and *Faecalibacterium* might play an important role in synthesis or degradation of fatty acyls and glycerophospholipids. *Comamonas* showed a significantly positive association with 14 amino acids, indicating a robust contribution of *Comamonas* to synthesis of amino acids (Fig. 7C). *Acinetobacter*, *Comamonas*, *Faecalibacterium*, *Sphingomonas*, and *Streptococcus* were significantly positively associated with 7, 7, 7, 5, and 6 carbohydrates, respectively, which suggested that *Acinetobacter*, *Comamonas*, *Faecalibacterium*, *Sphingomonas*, and *Streptococcus* might participate in synthesis of carbohydrates (Fig. 7D). The correlation analysis also showed that *Comamonas* and *Streptococcus* might play a role in the synthesis of nucleosides and nucleotides (Fig. 7D).

### Microbiota and metabolites associated with clinical features

**Fig. 8:**
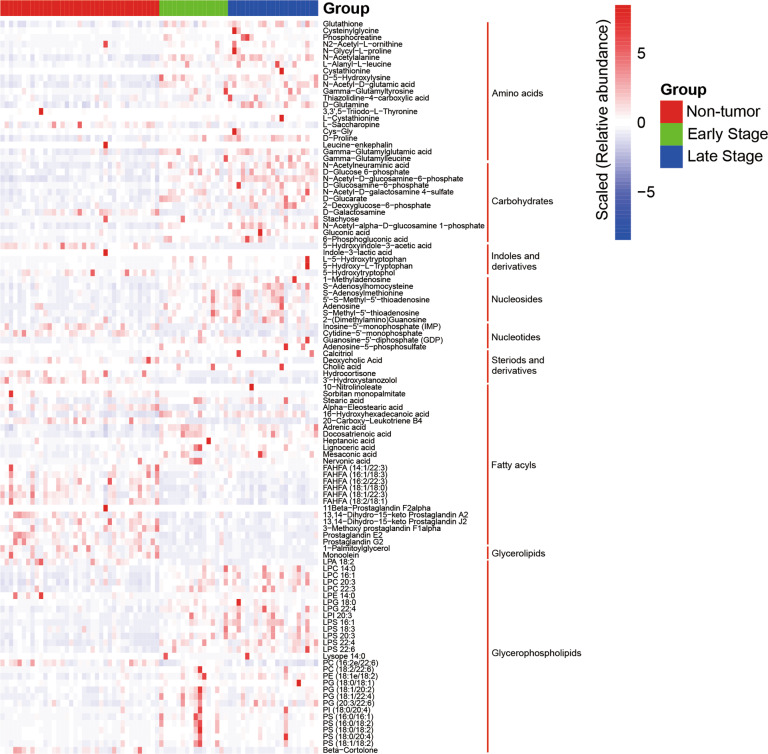
The heat map shows the association between metabolites and tumor stage. The differences in metabolites among non-tumor tissues (*n* = 37), early-stage (stage I–II, *n* = 16) and late-stage gastric tumor tissues (stage III, *n* = 21) were displayed. The heat map shows the scaled relative abundance (Lg) of 109 metabolites.

Cohort 1 for microbiome and untargeted metabolome analysis enrolled 16 early-stage (stage I–II) and 21 late-stage (stage III) GC patients (Table 1). The heat map showed that the majority of carbohydrates were gradually increased from non-tumor tissues to early-stage and late-stage tumor tissues (Fig. 8). Particularly, the concentration of *N*-acetyl-D-glucosamine-6-phosphate was gradually elevated from non-tumor tissues to early-stage and late-stage tumor tissues with significant difference (*Q* < 0.05, Fig. S1). A stepwise increase in the abundance of *Acinetobacter*, *Comamonas*, and *Sphingomonas* from non-tumor tissues to early-stage and late-stage tumor tissues was observed (Fig. S2D–F). Nevertheless, the differences were not significant (*Q* > 0.05). However, this trend was not observed for *Helicobacter*, *Lactobacillus*, and *Streptococcus* (Fig. S2A–C). As Figs. S3 and S4 show, no correlation was found between complications and microbiota, and between complications and metabolites.

## Discussion

In this study, the diversity and richness of gastric microbiota was found to be higher in tumor tissues than in non-tumor tissues, which is consistent with the result of a previous study [17]. However, Liu et al. observed decreased diversity and richness in peritumoral and tumoral tissues in comparison to non-tumor tissues from 276 GC patients [10]. There is no consensus on the relationship between microbial diversity and the mucosal tissues of stomach. The relative abundance of *Helicobacter* was reduced in GC tumor tissues compared to non-tumor tissues, which is consistent with results of two previous studies [10, 17]. The decrease of *Helicobacter* may be due to the loss of specialized glandular tissues and decreased acid secretion [10].

The abundance of *Lactobacillus* ranked second in the GC tumor tissues after the abundance of *Helicobacter* in this study, which differs from previous findings [10, 17]. However, several studies have found that the proportion of *Lactobacillus* was higher in GC patients compared to that found in healthy controls [8, 9, 18]. Liu et al. also found that *Lactobacillus* was higher in GC tumor tissues compared to that in non-tumor tissues [10]. Sonveaux et al. reported that *Lactobacillus* may produce metabolites that could be used as an energy source for tumor growth and angiogenesis [19]. Previous studies have also shown that the abundance of *Streptococcus* was increased in GC tumor tissues compared to non-tumor tissues [10, 17]. The abundance of *Streptococcus* was found to be elevated in tumor tissues from patients with lung cancer in comparison to non-tumor tissues [20]. *Streptococcus* displayed a correlation with upregulation of the ERK and PI3K signaling pathways in patients with lung cancer, and in vitro exposure of airway epithelial cells to *Streptococcus* led to upregulation of these same signaling pathways [21]. We found that *Bacteroides* exhibited higher abundance in the tumor tissues than in the non-tumor tissues. A restricted gastric microbiota containing only *Lactobacillus*, *Bacteroides*, and *Clostridium* promoted GC development at a similar rate to the complex microbiota in insulin-gastrin transgenic mice [22].

Our microbiome study shares some similarities with the two previous studies, but is also a little different from the two studies [10, 17]. The study by Shao et al. utilized gastric cardia tissues for microbiome analysis [17], while the study by Liu et al. used proximal stomach, body/fundus, and antrum tissues for research [10]. Our study also used proximal stomach, body/fundus, and antrum tissues. The difference between our study and the study by Liu et al. is that the samples of the study by Liu et al. were mainly from body/fundus, and antrum, while the samples of our study were primarily from proximal stomach, and antrum. Therefore, the differences between the three studies may result from samples with different tumor localization.

Our metabolome analysis of GC tumor tissues and the matched non-tumor tissues revealed 150 differential metabolites, including amino acids, carbohydrates and carbohydrate conjugates, fatty acyls, glycerophospholipids, nucleosides, and nucleotides. Consistent with previous studies [12, 13], the majority of the discriminative metabolites in the amino acid class displayed higher relative abundance in the tumor tissues than in the non-tumor tissues. Because tumor cells utilize amino acids to generate energy and synthesize proteins and nucleosides, increased concentrations of amino acids are essential for tumor cell proliferation. We also observed enhanced relative abundance of carbohydrates and carbohydrate conjugates in the tumor tissues in comparison to the non-tumor tissues. Elevated glucose uptake is a metabolic feature of tumor cells [23], and carbohydrates and carbohydrate conjugates may be used as a source of glucose by tumor cells. Thus, increased carbohydrates and carbohydrate conjugates may be vital in providing enough glucose to satisfy the energy requirements for tumor cell growth. Increased levels of nucleosides were found in the GC tumor tissues in this study, which is consistent with a previous study [12]. Kaji et al. reported that the concentrations of nucleosides were increased in GC patients with peritoneal recurrence compared with those without peritoneal recurrence. It is possible that enhanced levels of nucleosides, especially adenosine, may contribute to a shorter survival in GC patients. Adenosine is a key metabolic and immune-checkpoint regulator that participates in tumor escape from the host immune system [24]. Many therapies targeting adenosine metabolism are in progress. One study found that six glycerophospholipids positively associated with the risk of prostate cancer [25]. Interestingly, we observed 25 glycerophospholipids with increased levels in the GC tumor tissues, which may play important roles in GC development.

KEGG enrichment analysis showed that the pathway of glutathione, cysteine, and methionine metabolism contained 5 metabolites (glutathione, *S*-adenosylhomocysteine, *S*-adenosylmethionine, L-cystathionine, and *S*-methyl-5′-thioadenosine) with significantly increased relative abundance in the GC tumors. Kaji et al. reported that glutathione exhibited a higher level in GC tumor tissues than in the non-tumor tissues [12]. The level of glutathione was also found to be increased in tumor tissues from patients with chromophobe renal cell carcinoma in comparison to non-tumor tissues [26]. Glutathione and cysteine are very important antioxidants, and *S*-adenosylhomocysteine, *S*-adenosylmethionine, L-cystathionine, and *S*-methyl-5′-thioadenosine can serve as precursors of glutathione and cysteine. Therefore, the increased levels of metabolites in this pathway could provide GC tumor tissues with robust antioxidation ability. *Helicobacter* negatively associated with the discriminative metabolites in this pathway, whereas *Lactobacillus* and *Streptococcus* displayed a positive correlation with these metabolites, suggesting that *Helicobacter*, *Lactobacillus*, and *Streptococcus* were collectively responsible for the increased relative abundance of the differential metabolites in this pathway. Our data also showed that the levels of the discriminative metabolites in the pathway of fatty acid biosynthesis were upregulated by the collective activity of *Helicobacter, Faecalibacterium, Lactobacillus*, and *Bacteroides*. Consistently, we found that *Helicobacter*, *Lactobacillus, Faecalibacterium*, and *Bacteroides* might be collectively responsible for altered relative abundance of metabolites in the classes of fatty acyls and glycerophospholipids. The elevated relative abundance of the differential metabolites in the pathway of amino sugar and nucleotide sugar metabolism could be due to the collective influence of *Helicobacter*, *Lactobacillus*, *Streptococcus*, *Prevotella*, *Acinetobacter*, *Comamonas*, *Empedobacter*, and *Faecalibacterium*, which was in consistence with the observation that *Helicobacter*, *Lactobacillus*, *Acinetobacter*, *Comamonas*, *Faecalibacterium*, *Sphingomonas*, and *Streptococcus* might be collectively responsible for the synthesis of carbohydrates. *Helicobacter* and *Lactobacillus* were negatively and positively associated with the majority of differential metabolites in the classes of amino acids, carbohydrates, nucleosides, nucleotides, and glycerophospholipids, respectively, indicating that *Helicobacter* and *Lactobacillus* might contribute to degradation and synthesis of the majority of differential metabolites in these classes, respectively. These results indicated that the metabolome profiles of the GC tumor tissues were strongly influenced by *Helicobacter*, *Lactobacillus*, and other microorganisms, which might promote GC development.

Our study had several limitations. First, the sample size is very small, resulting in the lack of significant correlation between clinical features and microbiome, and between clinical features and metabolome. Second, we did not perform longitudinal studies since we could not obtain serial tissue samples from the recruited patients. Third, we utilized PLS-DA, which is highly susceptible to overfitting [27], to characterize the differential metabolites between GC tumor and non-tumor tissues. Thus, the proper model validation was required and we demonstrated that the model was not overfitted. Fourth, the diet could heavily influence both the gastric microbiota and metabolites, but we could not obtain the diet information of patients to analyze the effect of diet on gastric microbiome and metabolome.

In summary, for the first time, we profiled the microbiome and metabolome of tumor tissues and matched non-tumor tissues from GC patients. The diversity and composition of the gastric microbiota were significantly different between the tumor and non-tumor tissues. *Helicobacter* was enriched in the non-tumor tissues, while *Lactobacillus*, *Streptococcus*, *Acinetobacter*, *Prevotella*, and six additional genera were enriched in the tumor tissues. The metabolome profiles in the GC tumor tissues were significantly different from those in the matched non-tumor tissues, which may be partly due to the collective activities of *Helicobacter*, *Lactobacillus*, and other bacteria. The differences in gastric microbiome and metabolome profiles eventually affect GC carcinogenesis and progression. The functions of these microbiota and metabolites are worthy of further research as they may reveal or strengthen a GC therapy.

## Patients and methods

### Patients

Thirty-seven patients diagnosed with primary GC and undergoing gastrectomy between January 2018 and August 2019, at the First Affiliated Hospital, School of Medicine, Zhejiang University, were enrolled for microbiome and untargeted metabolome analysis. Twenty additional GC patients undergoing gastrectomy between June and August 2021, at The First Affiliated Hospital of Nanchang University, were recruited for targeted metabolome analysis to validate the putative metabolite biomarkers, 1-methylnicotinamide and *N*-acetyl-D-glucosamine-6-phosphate. All patients received general anesthesia before operation. Most of the patients underwent radical resection of GC, whereas a small number of patients underwent partial gastrectomy. The non-tumor tissue used as the control sample was the gastric mucosal tissue 5 cm away from the matched tumor tissue. Their general clinical data including age, gender, body mass index (BMI), and histories of hypertension and diabetes were recorded (Table 1). All GC patients were diagnosed by postoperative pathological examinations. The clinical pathological features of GC, such as tumor stage, tumor differentiation and Lauren type of tumor were recorded. The clinical staging was determined according to the 8th edition American Joint Committee on Cancer (AJCC) cancer staging manual of GC TNM Staging. The detailed exclusion criteria were described in supplementary methods and Fig. S5. The study was approved by the Ethics Committee of the First Affiliated Hospital, School of Medicine, Zhejiang University (2020-IIT-572), and the Medical Research Ethics Committee of the First Affiliated Hospital of Nanchang University (2021-9-001) as per the Declaration of Helsinki. Informed written consent was obtained from all patients before recruitment.

### DNA extraction, construction of amplicon library, and sequencing

The genomic DNA of GC tumor and non-tumor tissues was extracted using cetyltrimethylammonium bromide/sodium dodecyl sulfate method. DNA concentration and purity were obtained by Nanodrop 2000 Spectrophotometer (Thermo Scientific) and 1% agarose gel electrophoresis. DNA was diluted to 1 ng/μL using sterile water. V3-V4 region of 16s rRNA gene was amplified. The primers containing the barcode are 341F (CCTAYGGGRBGCASCAG) and 806R (GGACTACNNGGGTATCTAAT). All PCR reactions were performed in 30 μL volume, including 15 μL of Q5^®^ High-Fidelity 2X Master Mix (New England Biolabs, # M0492L), 0.2 μM of forward and reverse primers, and about 10 ng of template DNA or sterile water (negative control). PCR conditions: initial denaturation at 98 °C for 1 min, followed by 30 cycles of denaturation at 98°C for 10 s, annealing at 50°C for 30 s, and extension at 72 °C for 30 s, final extension at 72 °C for 5 min. PCR products were analyzed using 2% agarose gel electrophoresis. According to the manufacturer’s recommendation, the Ion Plus Fragment Library Kit 48 rxns (Thermo Scientific, # 4471252) was used to generate the sequencing library. The library quality was evaluated by the Qubit@ 2.0 Fluorometer (Thermo Scientific). At last, the library was sequenced on the Ion S5^TM^ XL platform to generate 400–600 bp single-end reads.

### Sequencing data analysis

Filter the raw reads following the Cutadapt quality control process to obtain the high-quality clean reads [28]. Chimera sequences were detected and removed using the UCHIME algorithm software [29]. Sequences with more than 97% similarity were allocated to one operational taxonomic unit (OTU) using Uparse software [30]. The taxonomic information was annotated by Silva database based on Mothur algorithm. OTU abundance information was normalized to the sample with the least sequences. Alpha diversity was analyzed using QIIME (Version 1.7.0) to investigate species diversity [31]. QIIME software (Version 1.7.0) was used to calculate the beta diversity based on the weighted Unifrac distance to evaluate differences in microbial community composition. Non-metric multidimensional scaling (NMDS) and principal coordinate analysis (PCoA) were performed. Nonparametric Kruskal-Wallis rank-sum test and the Wilcoxon matched-pairs signed rank test were used to perform linear discriminant analysis (LDA) effect size (LEfSe) analysis to detect discriminative taxa with significant difference between GC tumor and non-tumor tissues.

### Tissue sample preparation for untargeted metabolome analysis

Tissues (100 mg) were grounded with liquid nitrogen and the homogenate was resuspended with prechilled 80% methanol and 0.1% formic acid (FA) by well vortexing. Then the samples were incubated on ice for 5 min and were centrifuged at 15,000 rpm for 5 min (4 °C). The supernatant was diluted to a final concentration containing 53% methanol by LC-MS grade water. The samples were subsequently transferred to a fresh Eppendorf tube and then centrifuged at 15,000*g* for 10 min (4 °C). Finally, the supernatant was injected into the UHPLC-MS/MS system.

### UHPLC-MS/MS condition for untargeted metabolome analysis

UHPLC-MS/MS analysis was performed using a Vanquish UHPLC system coupled with an Orbitrap Q Exactive series mass spectrometer (Thermo Fisher). Samples were injected into a Hyperil Gold column at a flow rate of 0.2 mL/min. The eluents for the positive polarity mode were eluent A (0.1% FA in water) and eluent B (methanol).The eluents for the negative polarity mode were eluent A (5 mM ammonium acetate, pH 9.0) and eluent B (methanol).The solvent gradient was set as follows: 2% B, 1.5 min; 2-100% B, 12.0 min; 100% B, 14.0 min; 100-2% B, 14.1 min; 2% B, 17 min. The Q Exactive mass spectrometer worked under positive and negative polarity mode, the spray voltage was 3.2 kV, and the capillary temperature was 320 °C.

### The conditions for targeted metabolome

The mixed standard solution of *N*-acetyl-D-glucosamine-6-phosphate disodium salt (J&K Scientific) with a concentration of 5 mg/mL and 1-methylnicotinamide chloride (Sigma Aldrich) with a concentration of 2 mg/mL was prepared. The creatinine-d3 (Shanghai ZZBio) was used as the internal standard.

Tissues (50 mg) were grounded with liquid nitrogen and the homogenate was resuspended with 150 μL of prechilled 80% methanol including creatinine-d3 (200 ng/mL) by well vortexing. Then the samples were incubated on ice for 5 min and were centrifuged at 12,000 rpm for 10 min (4 °C). The supernatant was injected into the HPLC-MS/MS system.

HPLC-MS/MS analysis was performed using an ExionLC^TM^ AD HPLC system coupled with a QTRAP^®^ 6500 plus mass spectrometer (AB Sciex). Samples were injected into an ACQUITY UPLC HSS T3 column at a flow rate of 0.3 mL/min. The eluents for HPLC were eluent A (0.1% FA in water, 10 mM ammonium acetate) and eluent B (methanol). The solvent gradient was set as follows: 2% B, 1.0 min; 2-100% B, 1.5 min; 100% B, 2.0 min; 100-2% B, 2.1 min; 2% B, 3.0 min. A QTRAP^®^ 6500 plus mass spectrometer equipped with electrospray ionization source was operated in positive/negative ion mode with a spray voltage of 4500 V, and ion source temperature of 550 °C. The pressure of curtain gas, ion source gas 1 and 2 were 35, 60, and 60 psi, respectively.

The standard solution was diluted in gradient, and the standard solutions with different concentrations were detected by HPLC-MS. The standard curve was plotted with the concentration of the standard as the abscissa and the ratio of the peak area of the standard to that of the internal standard as the ordinate.

### Statistical analysis

The statistical analyses were performed using GraphPad Prism (Version 8.0; GraphPad Software) software. Statistical significance was defined as a two-sided *P* value of <0.05. The Wilcoxon matched-pairs signed rank test was used to calculate the difference in observed species, Shannon index, the abundance of taxa, and the concentration of metabolites between groups. *P* values were corrected using Benjamini-Hochberg method, and the corrected *P* values were denoted as *Q* values. Microbiome–metabolome correlation analysis was performed using Spearman’s correlation method and displayed using R software (Version 3.6.1).

## Supplementary information

Supplementary information.

Table S1

Table S2

Fig. S1

Fig. S2

Fig. S3

Fig. S4

Fig. S5

cddis-author-contribution-form

Agreement_Author_Change

Ethics statement-1

Ethics statement-2

Reproducibility Checklist

## Data Availability

Raw sequence data of 16s rRNA microbiome have been deposited in the China National Microbiological Data Center (Project accession number NMDC10017675 and microbiome accession numbers NMDC40001044 to NMDC40001117). The corresponding author has access to all data in the study.
