# Supplementary material for: Interactions between gastric microbiota and metabolites in gastric cancer
Source: Cell Death Dis. 2021 Nov 24;12(12):1104. doi: 10.1038/s41419-021-04396-y (PMC8613192; doi:10.1038/s41419-021-04396-y)
Supplement: Supplementary file 1 — Supplementary information. [file 41419_2021_4396_MOESM1_ESM.docx]

**Interactions between gastric microbiota and metabolites in gastric cancer**

**Daofeng Dai^1^, Yan Yang^2^, Jieqing Yu^1^, Tianfeng Dang^1^,** **Wenjing Qin^3^, Lisong Teng^2^, Jing Ye^1^, Hongqun Jiang^1^**

Correspondence: H Jiang (jianghq@ncu.edu.cn), Jing Ye (yjholly@email.ncu.edu.cn), L Teng (lsteng@zju.edu.cn), and D Dai (ddf2010@alumni.sjtu.edu.cn).

^1^Jiangxi Otorhinolaryngology Head and Neck Surgery Institute, Department of

Otorhinolaryngology-Head and Neck Surgery, The First Affiliated Hospital of Nanchang University, Nanchang, Jiangxi, China

^2^Department of Surgical Oncology, The First Affiliated Hospital, School of Medicine, Zhejiang University, Hangzhou, Zhejiang, China

^3^Human Genetic Resources Center, The First Affiliated Hospital of Nanchang University, Nanchang, Jiangxi, China

**Supplementary information**

**Table S1.** Quality control data of 16s rRNA gene sequencing.

**Table S2.** Differential metabolites identified by untargeted metabolome in this study.

**Fig. S1** The association between tumor stage and the relative abundance of two metabolites. **A, B** The relative abundance of N-Acetyl-D-glucosamine-6-phosphate **(A)** and N-Acetyl-alpha-D-glucosamine-1-phosphate **(B)** was compared among non-tumor tissues (n = 37), early-stage (stage I-II, n = 16) and late-stage (stage III, n = 21) tumor tissues, respectively. The Kruskal-Wallis test was performed. *Q* value, adjusted *P* value.

**Fig. S2** Analysis of differential genera among normal tissues, early-stage, and late-stage tumor tissues. **A-F** The relative abundance of *Helicobacter* **(A)**, *Lactobacillus* **(B)**, *Streptococcus* **(C)**, *Sphingomonas* **(D)**, *Acinetobacter* **(E)**, and *Comamonas* **(F)** was compared non-tumor tissues (n = 37), early-stage (stage I-II, n = 16) and late-stage (stage III, n = 21) tumor tissues, respectively. The Kruskal-Wallis test was performed. *Q* value, adjusted *P* value.

**Fig. S3** The association between complications and the relative abundance of gastric microbiota. **A-F** The relative abundance of *Helicobacter* **(A)**, *Lactobacillus* **(B)**, *Streptococcus* **(C)**, *Sphingomonas* **(D)**, *Acinetobacter* **(E)**, and *Comamonas* **(F)** was compared between tumor tissues from patients with complications (n=10) or without complications (n=27), respectively.

**Fig. S4** The heat map shows the differences in metabolites between tumor tissues from gastric cancer patients with or without complications. The differences in metabolites between tumor tissues from patients with complications (n=10) or without complications (n=27) were displayed. The heat map shows the scaled relative abundance (Lg) of 109 differential metabolites.

**Fig. S5** Flowchart explaining the enrollment of gastric cancer patients.

**Supplementary methods**

**Exclusion criteria of patients**

The exclusion criteria were as follows: gastrointestinal bleeding, peptic ulcer; hepatitis, liver cyst, pneumonia; pyloric obstruction; BMI＞30; history of malignancy or receive radiotherapy/chemotherapy before operation; recent (< 3 months prior) use of antibiotics, probiotics, prebiotics, symbiotics; gastric cancer with metastasis; no paired tissues (Fig. S5). One hundred and nineteen patients with primary GC were recruited, and sixty-two patients were excluded. Thirty-seven pairs of non-tumor and tumor tissues were used for microbiome and untargeted metabolome analysis. Twenty additional pairs of non-tumor and tumor tissues were utilized for targeted metabolome analysis. After gastrectomy, tissue samples were cut into small pieces (about 100 mg for each piece), rinsed with sterile water, and frozen in liquid nitrogen.

**Compound identification and annotation**

The raw data was preprocessed by Compound Discoverer 3.1 (Thermo Fisher) software. The data was screened by retention time and mass-to-charge ratio, and the peaks were aligned. Then, the accurate molecular weight of the compound was determined by the mass-to-charge ratio in the high-resolution extracted ion chromatogram, and the molecular formula was predicted according to the deviation of mass number and ion information of adduct. By matching with the fragment ion, collision energy and other information of each compound in the mzCloud database, the metabolites in samples were identified, and then the compounds with CV (coefficient of variation) less than 30% in QC (quality control) samples were selected as the final identification results for subsequent analysis. The characteristic peaks of molecules were matched and identified utilizing the high-quality mzCloud database constructed by the standard compound, mzVavel and MassList databases, which could identify as many metabolites in samples as possible and reflect the information of total metabolites to the greatest extent. These identified metabolites were annotated using Kyoto Encyclopedia of Genes and Genomes (KEGG) database, The Human Metabolome Database (HMDB), and lipidmaps database.

**Compound quantification and metabolome analysis**

The Compound Discoverer 3.1 was used to perform peak calibration, peak picking and quantification for each metabolite. Then the peak intensity was normalized to the total spectral intensity. Peaks were matched using mzCloud, mzVault and MassList databases to obtain accurately qualitative and relatively quantitative results. Principal components analysis (PCA) and Partial least-squares discriminant analysis (PLS‐DA) were performed at metaX. The metabolites with variable importance in projection (VIP) > 1 and *Q* value (adjusted *P* value) < 0.05 and fold change (FC) ≥ 2 or FC ≤ 0.5 were considered differential metabolites. The functions of these metabolites and metabolic pathways were studied using the KEGG database.

**Partial least squares discriminant analysis (PLS‐DA)**

PLS-DA was performed using “metaX” package in R software (version 3.4.3). The PLS-DA model of each comparison group was established and 7-fold cross-validation was performed to obtain the model evaluation parameters, namely R^2^Y and Q^2^Y. R^2^Y represents fitness of the model, while Q^2^Y indicates predicative ability of the model. The values of R^2^Y and Q^2^Y close to 1 indicate a stable and reliable model. PLS-DA is very susceptible to overfitting, so model validation is required.

**Model validation**

randomly scramble the grouping labels of each sample and then conduct modeling and prediction. Each modeling corresponds to a set of R^2^ and Q^2^. The values of R^2^ and Q^2^ obtained by 200 times of scramble and modeling can produce their regression lines. When the value of R^2^ is greater than that of Q^2^ and the intercept between Q^2^ regression line and Y axis is less than 0, the model is not overfitted. Two hundred permutations and two latent variables were required to fit the PLS-DA model.
