# Supplementary material for: Interactions between gastric microbiota and metabolites in gastric cancer
Source: Cell Death Dis. 2021 Nov 24;12(12):1104. doi: 10.1038/s41419-021-04396-y (PMC8613192; doi:10.1038/s41419-021-04396-y)
Supplement: Supplementary file 2 — Table S1 [file 41419_2021_4396_MOESM2_ESM.pdf]

Table S1. Quality control data of 16s rRNA gene sequencing

#Sample_name	Raw_reads (#)	Clean_Reads (#)	Base (nt)	AvgLen (nt)	Q20	GC%	Effective%
Non-tumor_01	83013	80330	31494649	392	81.5	48.54	96.77
Non-tumor_02	81969	80169	33043630	412	74.93	48.67	97.8
Non-tumor_03	85490	80071	30781014	384	74.29	48.7	93.66
Non-tumor_04	83053	80035	31462235	393	80.68	48.58	96.37
Non-tumor_05	87257	80161	32348391	403	85.1	50.11	91.87
Non-tumor_06	82591	80105	32327511	403	87.1	49.01	96.99
Non-tumor_07	82920	80212	31401402	391	81.64	48.6	96.73
Non-tumor_08	82226	80015	32291666	403	86.89	49.24	97.31
Non-tumor_09	88656	80173	33317176	415	72.02	50.49	90.43
Non-tumor_10	84963	80099	31406690	392	81.44	48.72	94.28
Non-tumor_11	84104	80104	31514125	393	79.35	48.83	95.24
Non-tumor_12	81811	80057	31172587	389	77.83	48.42	97.86
Non-tumor_13	84541	80251	31289418	389	79.17	48.52	94.93
Non-tumor_14	84759	80069	32294189	403	85.49	50.08	94.47
Non-tumor_15	82718	80091	32396533	404	86.63	49.46	96.82
Non-tumor_16	88747	80160	31952271	398	72.56	53.21	90.32
Non-tumor_17	85652	80060	30802622	384	74.78	48.68	93.47
Non-tumor_18	84097	80203	32579725	406	84.98	49.61	95.37
Non-tumor_19	84908	80094	31813433	397	77.64	48.97	94.33
Non-tumor_20	85071	80189	32493053	405	86.66	49.54	94.26
Non-tumor_21	82991	80033	32261513	403	87.35	49.06	96.44
Non-tumor_22	81764	80109	32311193	403	86.95	49.1	97.98
Non-tumor_23	81965	80043	32285024	403	86.22	49.14	97.66
Non-tumor_24	84874	80142	30868548	385	73.45	48.42	94.42
Non-tumor_25	85371	80101	32742724	408	86.14	49.74	93.83
Non-tumor_26	86255	80191	31259283	389	71.35	50.64	92.97
Non-tumor_27	86146	80090	33303352	415	88.19	50.54	92.97
Non-tumor_28	87038	80189	33093266	412	84.14	50.38	92.13
Non-tumor_29	81942	80043	32527887	406	89.4	49.14	97.68
Non-tumor_30	82306	80198	33337475	415	86.38	52.89	97.44
Non-tumor_31	82439	80213	32599573	406	88.96	49.36	97.3
Non-tumor_32	84639	80111	32609820	407	87.78	49.2	94.65
Non-tumor_33	74331	72257	30690975	424	88.01	54.35	97.21
Non-tumor_34	86241	80169	33535703	418	88.63	52.31	92.96
Non-tumor_35	72743	69150	28616998	413	88.42	52.64	95.06
Non-tumor_36	83543	80204	33521289	417	88.15	51.63	96
Non-tumor_37	88795	80103	33125025	413	88.85	53.17	90.21
Tumor_01	83565	80310	31954551	397	70.42	50.09	96.1
Tumor_02	85890	80097	32193439	401	74.22	50.09	93.26
Tumor_03	81961	80145	31161355	388	73.87	50.05	97.78
Tumor_04	85942	80111	32654163	407	66.28	52.74	93.22
Tumor_05	84225	80050	31245386	390	71.45	50.31	95.04
Tumor_06	83616	80079	32222890	402	76	54.95	95.77
Tumor_07	65524	59716	23546906	394	70.19	50.43	91.14
Tumor_08	84122	80192	32101057	400	69.41	50.53	95.33
Tumor_09	81863	80137	32153577	401	71.86	50.76	97.89
Tumor_10	88031	80151	32930591	410	79.18	51.34	91.05
Tumor_11	83686	80153	32625393	407	81.07	50.42	95.78
Tumor_12	87863	80148	32638106	407	83.11	51.01	91.22
Tumor_13	74862	71302	29152660	408	75.72	51.07	95.24
Tumor_14	83787	80063	32210557	402	76.95	55.91	95.56
Tumor_15	83303	80171	33204838	414	81.66	51.27	96.24
Tumor_16	73212	69353	27004822	389	66.64	50.78	94.73
Tumor_17	84524	80029	33388977	417	86.8	53.29	94.68
Tumor_18	88079	80235	33263021	414	80.76	51.19	91.09

Tumor_19	85529	80211	33618371	419	87.07	52.31	93.78
Tumor_20	88661	80114	33257315	415	87.05	51.92	90.36
Tumor_21	83452	80074	33714287	421	87.43	51.78	95.95
Tumor_22	85574	80173	33099592	412	86.98	53.1	93.69
Tumor_23	88251	80110	33660631	420	86.65	52.19	90.78
Tumor_24	84289	80166	33728360	420	87.89	52.32	95.11
Tumor_25	87275	80067	33413777	417	86.59	53.26	91.74
Tumor_26	85803	80128	32877382	410	87.55	53.12	93.39
Tumor_27	87374	80102	31769187	396	69.15	51.31	91.68
Tumor_28	88916	80032	32251594	402	69.83	50.22	90.01
Tumor_29	84784	80031	33286204	415	80.16	51.88	94.39
Tumor_30	88879	80101	32929508	411	81.63	51.57	90.12
Tumor_31	87364	80227	32346798	403	73.7	51.55	91.83
Tumor_32	83868	80135	32857317	410	80.97	51.03	95.55
Tumor_33	88108	84276	34386053	408	71.67	52.64	95.65
Tumor_34	61351	59585	23861155	400	65.7	52.24	97.12
Tumor_35	84305	80151	33452612	417	82.69	51.03	95.07
Tumor_36	86425	80076	30695119	383	67.47	49.6	92.65
Tumor_37	73083	70051	28342796	404	66.52	51.56	95.85
