# Supplementary material for: Interactions between gastric microbiota and metabolites in gastric cancer
Source: Cell Death Dis. 2021 Nov 24;12(12):1104. doi: 10.1038/s41419-021-04396-y (PMC8613192; doi:10.1038/s41419-021-04396-y)
Supplement: Supplementary file 3 — Table S2 [file 41419_2021_4396_MOESM3_ESM.pdf]

Table S2. Differential metabolites identified by untargeted metabolome in this study

Metabolite name	log2FC	*P* -value	*Q* -value	ROC	VIP	Up/Down
Lignoceric acid	1.148903429	9.77E-09	2.55E-07	0.894083272	1.907818417	up
Adrenic acid	1.182473931	4.32E-08	8.93E-07	0.913805698	1.89343938	up
Stearic acid	1.138886971	4.95E-08	1.00E-06	0.833455077	1.808885507	up
N2-Acetyl-L-ornithine	1.035420976	9.82E-07	1.29E-05	0.855368882	1.661163565	up
S-Adenosylhomocysteine	1.244816021	2.05E-06	2.41E-05	0.804967129	1.618733767	up
L-Saccharopine	-1.064434393	7.23E-06	7.46E-05	0.778670562	1.65013284	down
Nervonic acid	1.132720181	0.002317373	0.009507577	0.69101534	1.278751895	up
5-Hydroxyindole-3-acetic acid	-3.146530775	5.35E-17	6.41E-14	0.946676406	2.491879921	down
1-Methylnicotinamide	1.894951049	3.03E-16	1.81E-13	0.956902849	2.488855232	up
Coproporphyrin III tetramethyl ester	-2.548082287	7.30E-16	2.92E-13	0.940832725	2.487806526	down
N-Acetyl-D-glucosamine 6-phosphate	1.293038407	9.77E-16	2.93E-13	0.951059167	2.396091382	up
Shikimic acid	-2.345062775	2.22E-15	5.31E-13	0.941563185	2.472074987	down
Xanthurenic acid	-1.542228548	1.85E-14	3.69E-12	0.930606282	2.368706291	down
20-Carboxy-Leukotriene B4	-1.700081952	4.31E-14	7.37E-12	0.929875822	2.394264204	down
3-Ureidopropionic acid	1.308324759	1.26E-13	1.89E-11	0.928414901	2.283858779	up
N-Acetyl-D-glutamic acid	1.389199865	4.17E-13	5.54E-11	0.926223521	2.242430455	up
N-Acetyl-alpha-D-glucosamine 1-phosphate	1.271859034	1.91E-12	2.29E-10	0.926223521	2.201397554	up
N-Acetylalanine	1.273729172	3.02E-12	3.15E-10	0.915266618	2.184959756	up
Kynurenine	2.619204367	3.15E-12	3.15E-10	0.937910884	2.243176903	up
D-5-Hydroxylysine	1.058678331	8.18E-12	7.54E-10	0.933528123	2.225906679	up
16-Hydroxyhexadecanoic acid	1.171952679	2.85E-11	2.44E-09	0.900657414	2.11150491	up
N-Acetylneuraminic acid	1.464204674	4.23E-11	3.38E-09	0.894083272	2.092495706	up
LPC 16:1	1.674510467	9.12E-11	6.83E-09	0.88604821	2.08112787	up
3'-Hydroxystanozolol	-2.039041274	1.13E-10	7.96E-09	0.888970051	2.188145391	down
Monoolein	-2.696052781	1.94E-10	1.29E-08	0.880934989	2.079631485	down
3-Methoxy prostaglandin F1alpha	-1.554233492	3.18E-10	1.91E-08	0.892622352	2.070948453	down
5-Hydroxy-L-Tryptophan	2.732609714	3.18E-10	1.91E-08	0.928414901	2.100060545	up
ACar 21:3	-1.876538184	5.76E-10	3.00E-08	0.869978086	2.019224865	down
N1-cyclooctyl-4-hydroxy-1-piperidinecarbothioamide	-3.00811691	1.36E-09	6.53E-08	0.905040175	2.183846723	down
1-(3-phenylpropanoyl)-4-piperidinecarboxylic acid	-1.143296721	1.60E-09	7.35E-08	0.863403944	2.190310083	down
D-Proline	1.052148159	1.78E-09	7.92E-08	0.880204529	1.96044255	up
Alpha-Eleostearic acid	-1.056352323	2.24E-09	9.60E-08	0.855368882	2.006808746	down
Adenosine	1.84066374	2.82E-09	1.17E-07	0.868517166	1.934837713	up
LPS 16:1	2.322211978	4.07E-09	1.53E-07	0.850255661	2.027514518	up
Sorbitan monopalmitate	-2.292430611	4.23E-09	1.54E-07	0.867786706	1.965555386	down
N-glycyl-L-proline	1.462838228	4.65E-09	1.59E-07	0.928414901	1.93654399	up
6-Methoxyquinoline N-oxide	-1.226535201	5.13E-09	1.65E-07	0.891891892	2.05288427	down
LPS 20:3	1.385614947	5.23E-09	1.65E-07	0.859021183	1.912281306	up
2-(Formylamino)Benzoic Acid	1.268120915	5.98E-09	1.84E-07	0.913805698	1.949121566	up
N-Acetyl-D-galactosamine 4-sulfate	1.325742662	6.61E-09	1.98E-07	0.850986121	1.914580733	up
1,4-dihydroxyheptadec-16-en-2-yl acetate	-3.98696368	6.95E-09	2.03E-07	0.866325785	1.973313114	down
LPS 22:4	1.102955363	7.49E-09	2.14E-07	0.850255661	1.897826962	up
Deoxycholic Acid	-1.287169289	8.76E-09	2.39E-07	0.859021183	1.917665016	down
LPC 22:3	1.23426137	9.77E-09	2.55E-07	0.861943024	1.890940262	up
(2S)-2-(2-hydroxypropan-2-yl)-2H,3H,7H-furo[3,2-g]chromen-7-one	1.958316475	1.04E-08	2.64E-07	0.853907962	1.915353354	up
Cytidine-5'-monophosphate	-1.230506327	1.23E-08	3.08E-07	0.860482104	2.058887759	down
2-Methylbutyl-beta-D-glucopyranoside	-2.75113647	1.28E-08	3.12E-07	0.888239591	2.154203545	down
2-Amino-1,3-octadecanediol	1.928530476	1.58E-08	3.78E-07	0.856829803	1.948500885	up
Guanosine-5'-diphosphate (GDP)	2.12619305	1.67E-08	3.91E-07	0.855368882	1.900878814	up

Docosatrienoic acid	1.377557131	2.48E-08	5.62E-07	0.940102264	2.11559559	up
1-Methyladenosine	1.3993751	2.98E-08	6.42E-07	0.850986121	1.858614333	up
5,7-dimethyl-2-phenylpyrazolo[1,5-a]pyrimidine	-1.58351358	3.00E-08	6.42E-07	0.84660336	2.020352649	down
L-beta-Imidazolelactic acid	1.192926308	3.27E-08	6.87E-07	0.84733382	1.828732563	up
Glycerophospho-N-palmitoyl ethanolamine	-1.001887782	7.29E-08	1.41E-06	0.867056245	1.86680348	down
Nicotinic Acid	1.097697507	7.70E-08	1.46E-06	0.864134405	1.851040325	up
FAHFA (16:2/22:3)	-1.502757524	8.99E-08	1.68E-06	0.833455077	1.833686103	down
Prostaglandin G2	-2.024457249	1.09E-07	1.98E-06	0.831263696	1.796675669	down
L-Kynurenine	2.731816675	1.18E-07	2.12E-06	0.850255661	1.910420644	up
6-methyl-4-(morpholinomethyl)-2H-chromen-2-one	1.15520049	1.42E-07	2.50E-06	0.829802776	1.894370632	up
2,3-dihydroxypropyl 12-methyltridecanoate	-2.17961773	1.54E-07	2.67E-06	0.840029218	1.878175622	down
Cystathionine	2.341013991	2.29E-07	3.92E-06	0.902848795	1.793177175	up
LPS 22:6	1.095503206	2.44E-07	4.00E-06	0.818845873	1.741606873	up
2-Arachidonoyl glycerol	-1.548356996	3.03E-07	4.90E-06	0.833455077	1.745896449	down
1-Palmitoylglycerol	-2.028796231	3.50E-07	5.52E-06	0.814463112	1.813620013	down
L-5-Hydroxytryptophan	2.322764422	4.31E-07	6.70E-06	0.8458729	1.818170471	up
PE (18:1e/18:2)	1.102867537	4.50E-07	6.92E-06	0.812271731	1.703073551	up
LPC 14:0	1.486972738	4.63E-07	7.02E-06	0.828341855	1.735434511	up
γ-Glutamylglutamic acid	1.108026382	5.49E-07	8.13E-06	0.810810811	1.699631296	up
5-Hydroxytryptophol	-1.762550427	5.84E-07	8.53E-06	0.80861943	1.926140006	down
13,14-Dihydro-15-keto Prostaglandin J2	-1.19011524	7.28E-07	1.03E-05	0.813002191	1.74380583	down
LPC 20:3	1.089014447	8.33E-07	1.15E-05	0.811541271	1.699887801	up
Andrographolide	-1.610238314	8.93E-07	1.22E-05	0.837837838	1.684117473	down
ACar 21:2	-1.406730872	9.35E-07	1.26E-05	0.799123448	1.65908878	down
PG (18:1/22:4)	1.916595575	9.65E-07	1.28E-05	0.80715851	1.659045342	up
LPG 22:4	1.395878222	1.65E-06	2.03E-05	0.813732652	1.640492371	up
FAHFA (18:2/18:1)	-1.196706247	1.65E-06	2.03E-05	0.842951059	1.756106955	down
PC (16:2e/22:6)	-1.179697011	1.66E-06	2.03E-05	0.836376917	1.648852286	down
FAHFA (18:1/22:3)	-1.033666504	2.01E-06	2.38E-05	0.804967129	1.696187195	down
PS (16:0/16:1)	1.770273226	2.72E-06	3.11E-05	0.804236669	1.61270931	up
Prostaglandin E2	-1.489846667	6.19E-06	6.57E-05	0.787436085	1.582228625	down
SM (d14:2/18:0)	1.134305429	7.16E-06	7.46E-05	0.784514244	1.549128596	up
D-Galactosamine	-1.432193491	7.70E-06	7.80E-05	0.796932067	1.569949722	down
Hydrocortisone	-1.634758013	8.75E-06	8.66E-05	0.766983199	1.704819936	down
D-(-)-Glutamine	1.079233921	1.55E-05	0.000145826	0.80715851	1.509870193	up
Beta-Cortolone	-1.054170835	1.84E-05	0.000169474	0.777940102	1.484397553	down
D-Sphingosine	1.542375065	1.92E-05	0.000175685	0.766983199	1.653550072	up
ACar 23:3	-1.513478305	2.17E-05	0.000193453	0.810080351	1.51056486	down
13,14-Dihydro-15-keto Prostaglandin A2	-1.319579969	2.23E-05	0.000193453	0.773557341	1.49293737	down
LPG 18:0	1.260364455	3.17E-05	0.000259961	0.783783784	1.535630046	up
2-deoxyglucose-6-phosphate	1.580239841	3.73E-05	0.000302266	0.761139518	1.49006546	up
PS (18:0/20:4)	2.187752115	4.40E-05	0.000351572	0.782322863	1.464555053	up
PS (18:1/18:2)	1.417441174	4.54E-05	0.000357646	0.786705625	1.421339529	up
Cys-Gly	1.483783905	5.05E-05	0.000392837	0.820306793	1.457012333	up
Oleoyl ethanolamide	-1.060742787	5.24E-05	0.000405003	0.755295836	1.427562578	down
PC (18:2/22:6)	1.56088531	5.34E-05	0.000409934	0.779401023	1.411767238	up
Anthranilic acid	1.384213009	5.57E-05	0.000424736	0.84441198	1.44631568	up
Leucine-enkephalin	-2.331074976	6.12E-05	0.000458893	0.818115413	1.599822556	down
Calcitriol	1.541230111	7.83E-05	0.000565162	0.756756757	1.383865965	up
LPS 18:3	1.0248213	7.93E-05	0.000569213	0.774287801	1.376432091	up
LPA 18:2	-1.210301715	0.000132893	0.000909746	0.734112491	1.663816126	down
Gluconic acid	1.392242434	0.000155331	0.001041575	0.799853908	1.345246786	up

Inosine-5'-monophosphate (IMP)	-1.671732094	0.000155707	0.001041575	0.763330898	1.422024827	down
L-Alanyl-L-leucine	1.06951832	0.000163892	0.001078806	0.733382031	1.329731776	up
Phosphocreatine	2.140597468	0.000177732	0.001163514	0.728268809	1.32708791	up
Gamma-Glutamylleucine	1.420164723	0.000184303	0.001193486	0.749452155	1.45001566	up
LPI 20:3	1.535575101	0.000202095	0.001281006	0.742878013	1.3486202	up
Stachyose	1.06815375	0.000210501	0.001313436	0.780861943	1.347718279	up
N1-(4-{[(ethylamino)carbothioyl]amino}phenyl)-2-hydroxybenzamide	-2.301823961	0.000251427	0.001513617	0.723155588	1.300712207	down
Heptanoic acid	1.094728191	0.000252887	0.001514794	0.772826881	1.290661963	up
PS (18:0/18:2)	1.306393049	0.000256006	0.001525848	0.756026297	1.307951853	up
D-Glucosamine 6-phosphate	1.463229063	0.000267518	0.001569657	0.752373996	1.281145411	up
10-Nitrolinoleate	1.492525912	0.000284376	0.001637895	0.759678598	1.330491186	up
PG (18:0/18:1)	1.303719825	0.000324861	0.001827151	0.788166545	1.277354568	up
PS (16:0/18:2)	1.667231521	0.000376985	0.002071691	0.740686633	1.256865562	up
S-Methyl-5'-thioadenosine	1.205522027	0.000381352	0.002086118	0.739956172	1.289974555	up
N1-Acetylspermine	2.293653179	0.000433932	0.002310449	0.766252739	1.280859813	up
PG (18:1/20:2)	1.047750297	0.000443326	0.00235002	0.745799854	1.295349326	up
2-(Dimethylamino)Guanosine	1.076453225	0.000481578	0.002541541	0.744338934	1.244195686	up
FAHFA (16:1/18:3)	-1.195913383	0.000500993	0.002632409	0.737764792	1.443143999	down
3-Phenyllactic acid	-1.013761439	0.00065487	0.003255328	0.88604821	1.350902054	down
PI (18:0/20:4)	1.744916538	0.000668221	0.003296404	0.737034332	1.202950894	up
L-(-)-3-Phenyllactic acid	-1.216868545	0.000668636	0.003296404	0.88604821	1.337911518	down
Methylimidazoleacetic acid	1.382728357	0.001283431	0.005892221	0.715120526	1.198875863	up
D-Glucose 6-phosphate	1.123113587	0.001286464	0.005892221	0.701241782	1.165079769	up
L-Cystathionine	1.736438145	0.001288616	0.005892221	0.700511322	1.168410263	up
Cysteinylglycine	1.346294	0.001436733	0.006422412	0.719503287	1.146670876	up
Gamma-Glutamyltyrosine	1.447651756	0.001544274	0.006877471	0.710007305	1.212632279	up
5'-S-Methyl-5'-thioadenosine	1.651900963	0.001575983	0.006966893	0.750182615	1.165486657	up
11beta-Prostaglandin F2alpha	-3.245478895	0.001757256	0.007655245	0.745069394	1.345951219	down
Hydrocinnamic acid	1.869218042	0.002020653	0.008645507	0.772826881	1.109071862	up
Cholic acid	2.934301375	0.002030765	0.008657853	0.742878013	1.124779192	up
A-Ketoglutaric Acid	1.257987434	0.002073011	0.008806622	0.707085464	1.235958496	up
FAHFA (18:1/18:0)	-1.066380786	0.002312943	0.009507577	0.818845873	1.385177796	down
D-Glucarate	1.254413562	0.002379375	0.00963004	0.708546384	1.21682882	up
6-Phosphogluconic acid	2.261563822	0.002563535	0.010203038	0.718042367	1.150274736	up
glutathione	1.107829574	0.002810245	0.011074583	0.783783784	1.067304621	up
S-Adenosylmethionine	1.63184184	0.003431521	0.012968336	0.734842951	1.065140188	up
Adenosine5-phosphosulfate	1.738666036	0.003835028	0.014030717	0.702702703	1.12353083	up
1-[4-(1-adamantyl)phenoxy]-3-piperidinopropan-2-ol hydrochloride	-1.797926405	0.00434065	0.015476483	0.710007305	1.025284518	down
FAHFA (14:1/22:3)	-1.199699678	0.005695454	0.019439413	0.661066472	1.131107324	down
LPE 14:0	-1.150763716	0.006031794	0.020470508	0.672753835	1.046900719	down
PG (20:3/22:6)	1.039941101	0.006747754	0.022409452	0.665449233	1.001623215	up
Lysope 14:0	1.681956597	0.007131781	0.02347218	0.689554419	1.153902997	up
N1-(4-chlorophenyl)-2-cyano-4,4-dimethyl-3-oxopentanamide	1.123841531	0.00771462	0.0250194	0.666910153	1.040761551	up
Riboflavin B2	-1.211630766	0.007727194	0.0250194	0.674214755	1.019825374	down
N1-(2,6-dimethylphenyl)-2-morpholinoacetamide	-1.306769301	0.01016141	0.03129401	0.688093499	1.043046037	down
mesaconic acid	1.149488762	0.011014328	0.033405481	0.649379109	1.017172556	up
Indole-3-lactic acid	-1.881704768	0.011595854	0.034992024	0.775018262	1.028485158	down
Thiazolidine-4-carboxylic acid	1.080556782	0.011801891	0.035435253	0.666910153	1.124378653	up
3,3',5-Triiodo-L-Thyronine	-1.112292919	0.01703795	0.047690336	0.660336012	1.177789914	down

Q -value, corrected *P* -value.
