# Supplementary figures and images for: Interactions between gastric microbiota and metabolites in gastric cancer
Source: Cell Death Dis. 2021 Nov 24;12(12):1104. doi: 10.1038/s41419-021-04396-y (PMC8613192; doi:10.1038/s41419-021-04396-y)

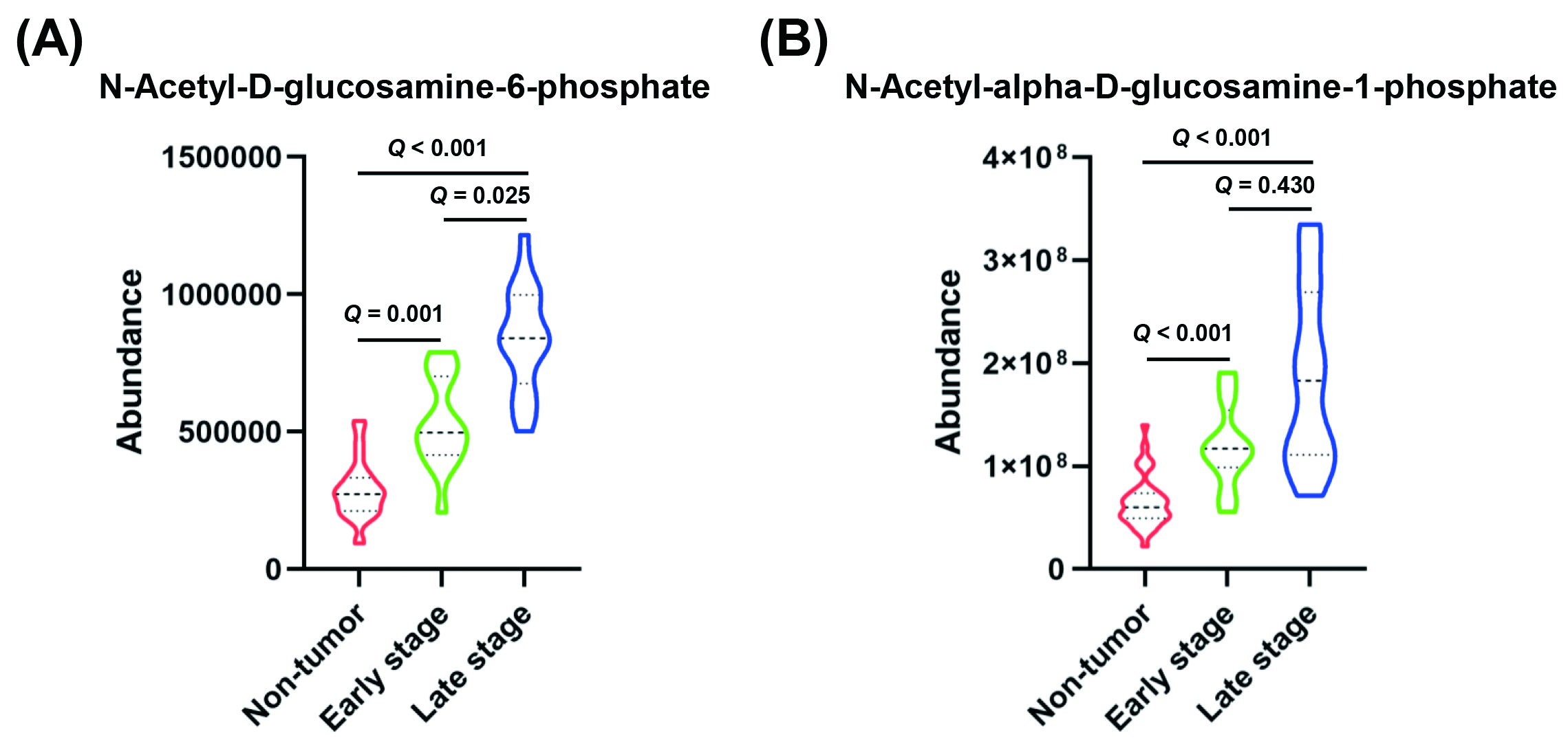

Supplement: Supplementary file 4 — Fig. S1 [file 41419_2021_4396_MOESM4_ESM.tif]

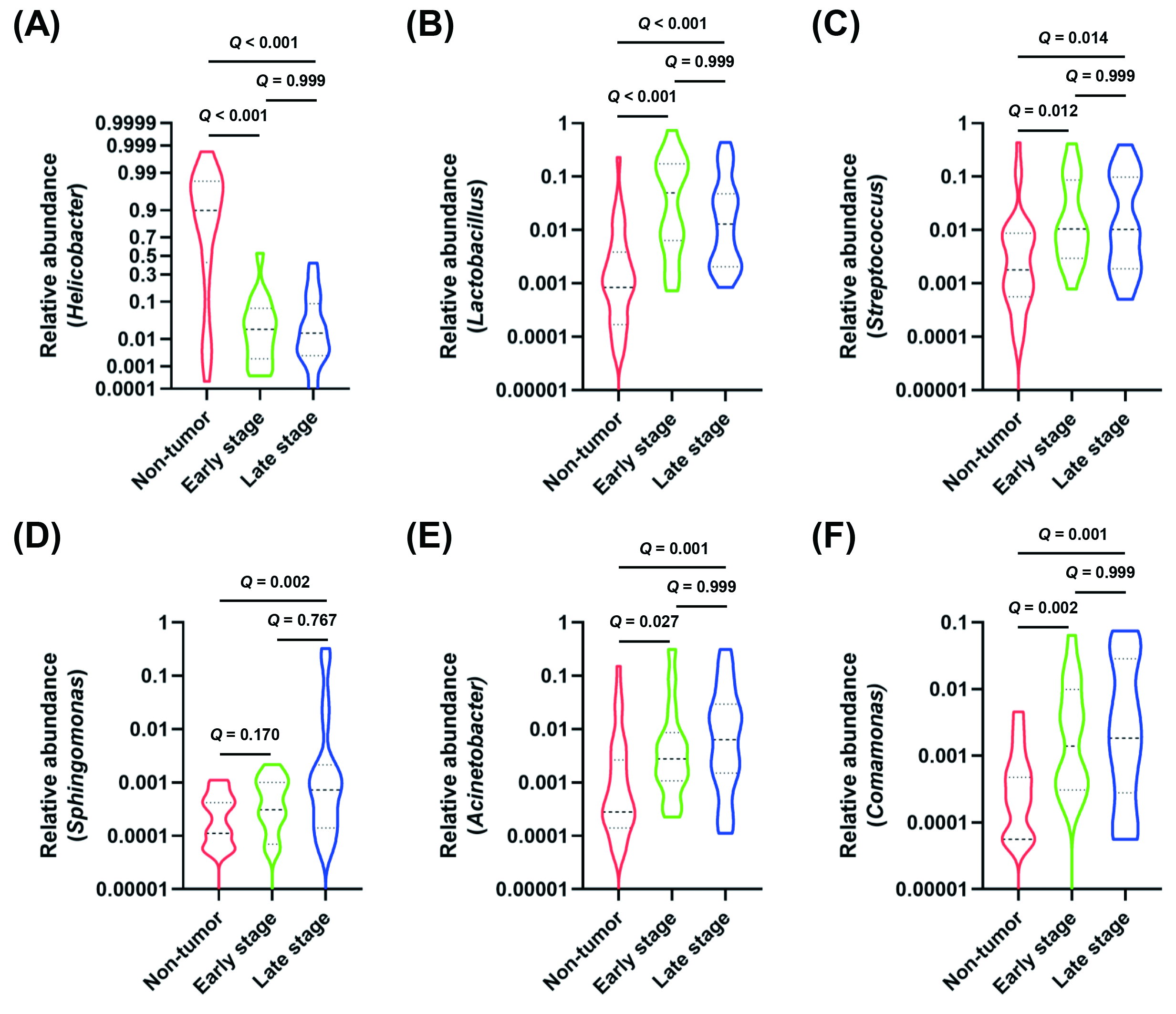

Supplement: Supplementary file 5 — Fig. S2 [file 41419_2021_4396_MOESM5_ESM.tif]

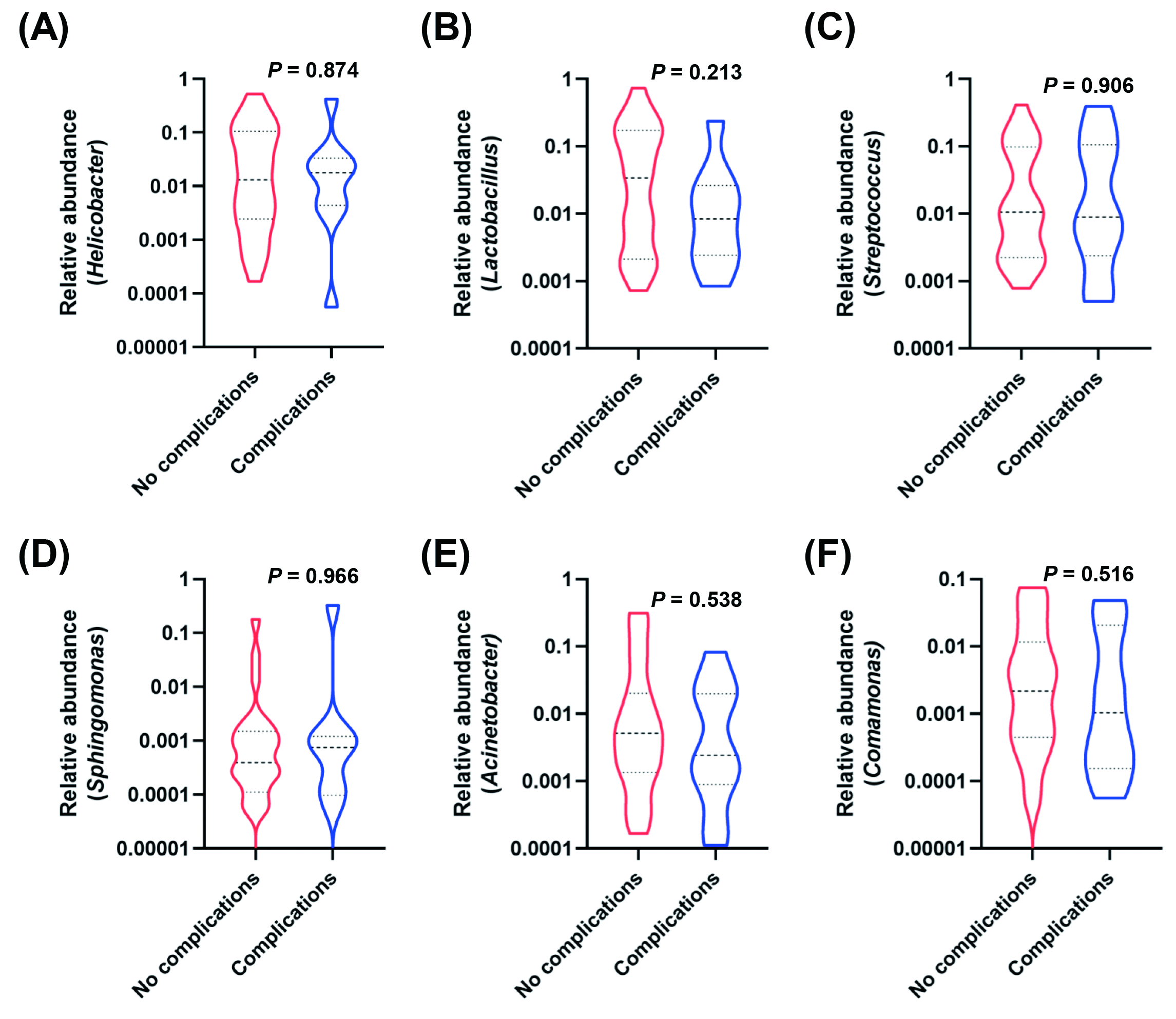

Supplement: Supplementary file 6 — Fig. S3 [file 41419_2021_4396_MOESM6_ESM.tif]

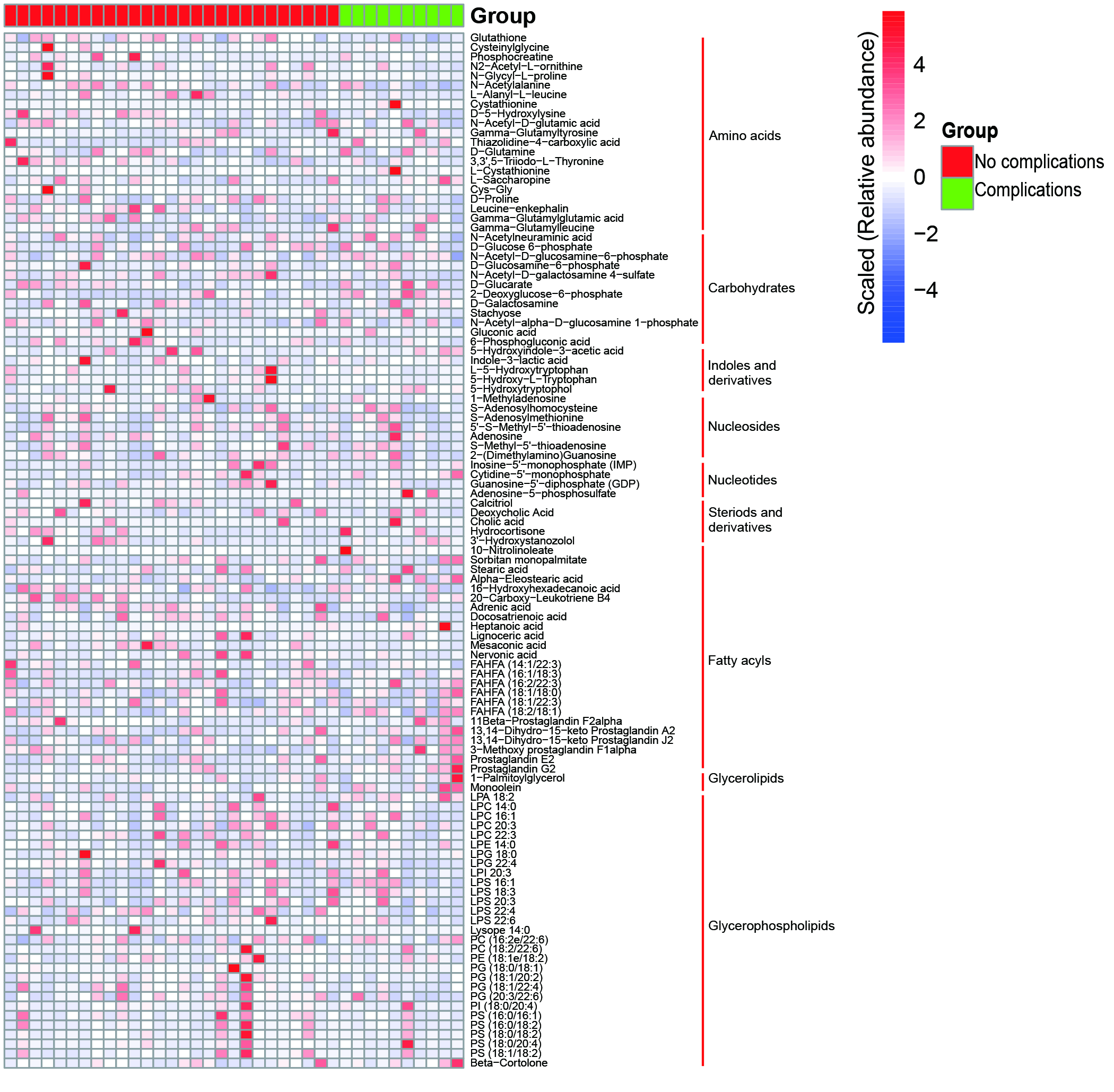

Supplement: Supplementary file 7 — Fig. S4 [file 41419_2021_4396_MOESM7_ESM.tif]

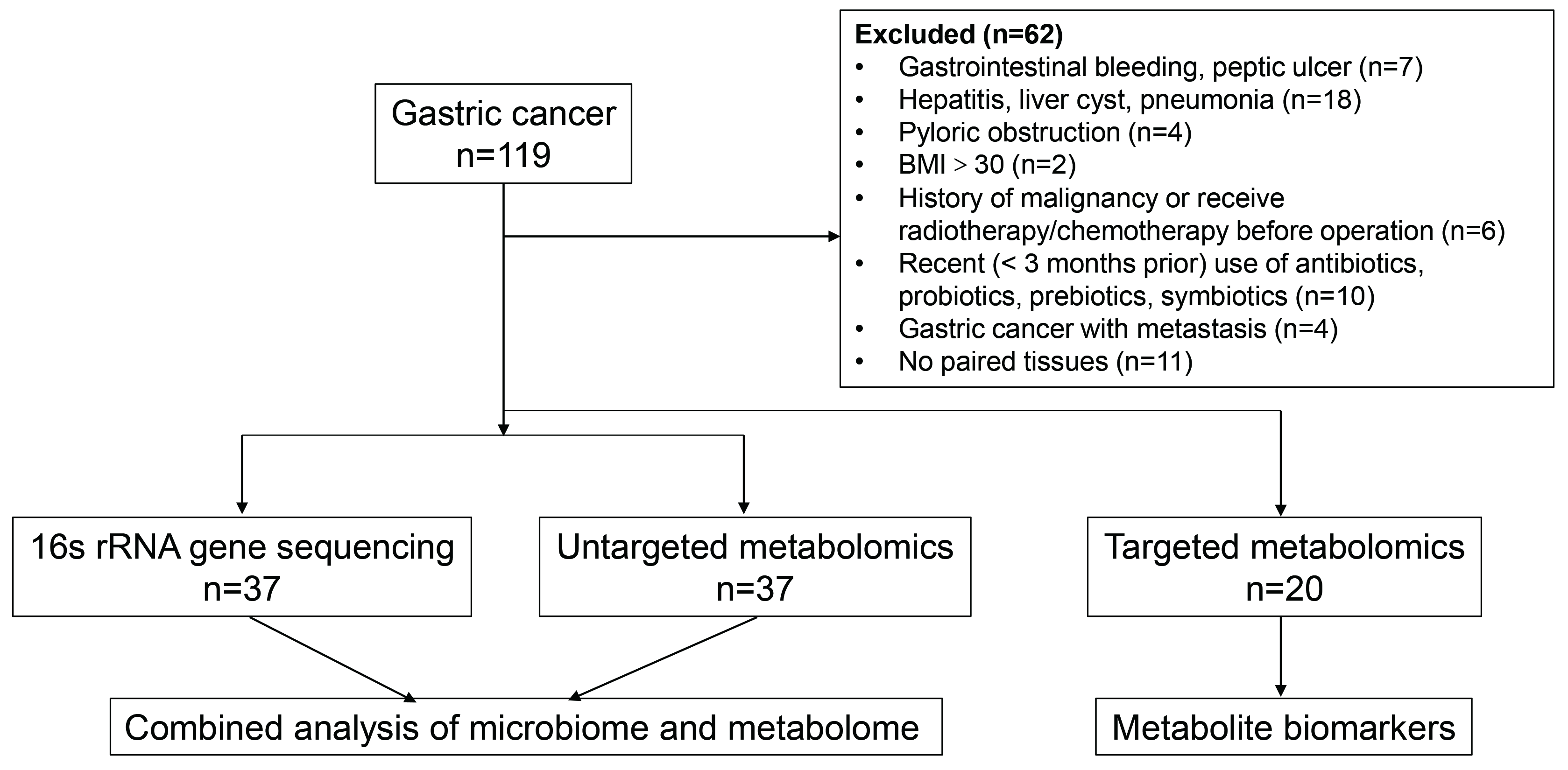

Supplement: Supplementary file 8 — Fig. S5 [file 41419_2021_4396_MOESM8_ESM.tif]

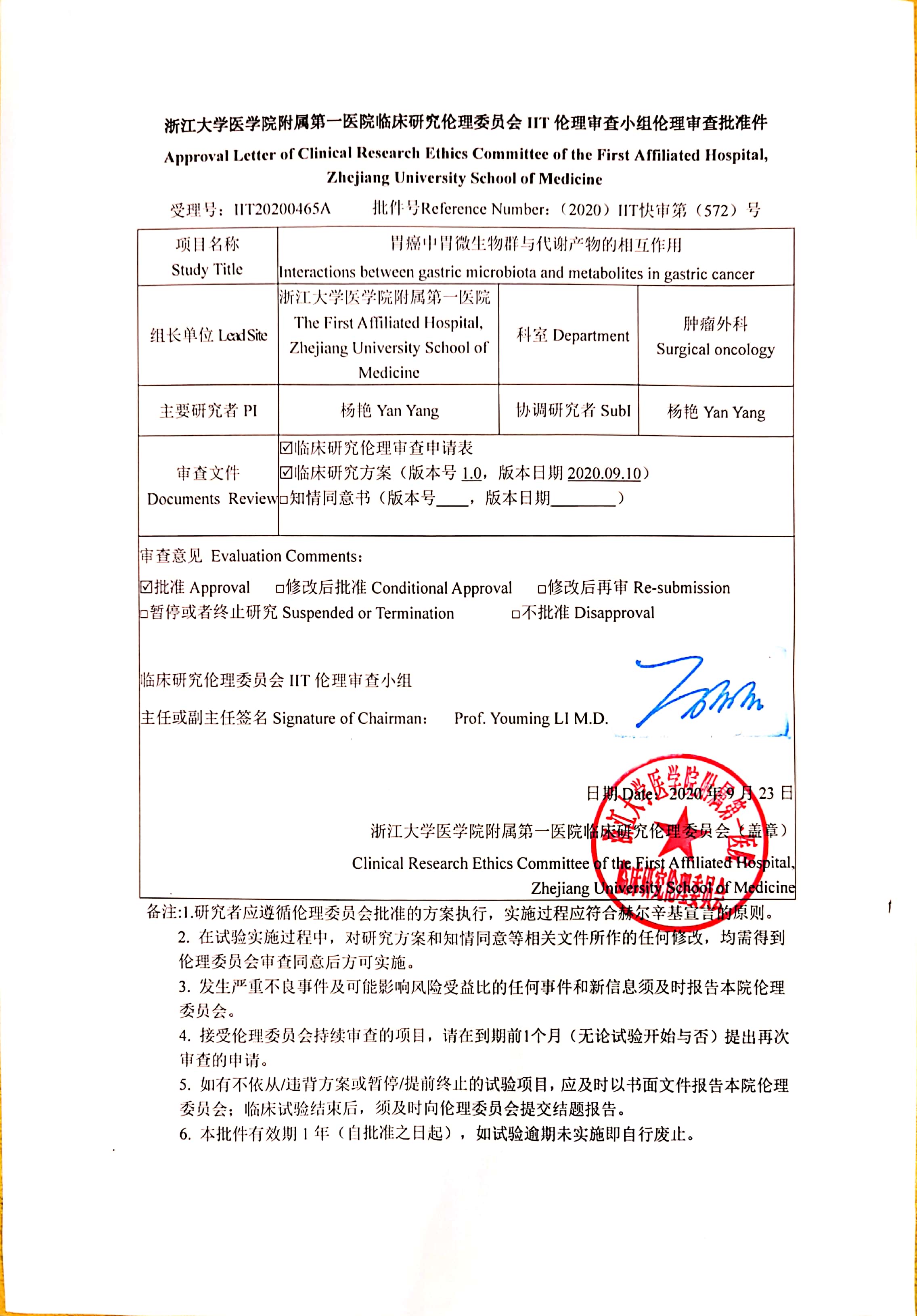

Supplement: Supplementary file 11 — Ethics statement-1 [file 41419_2021_4396_MOESM11_ESM.jpg]

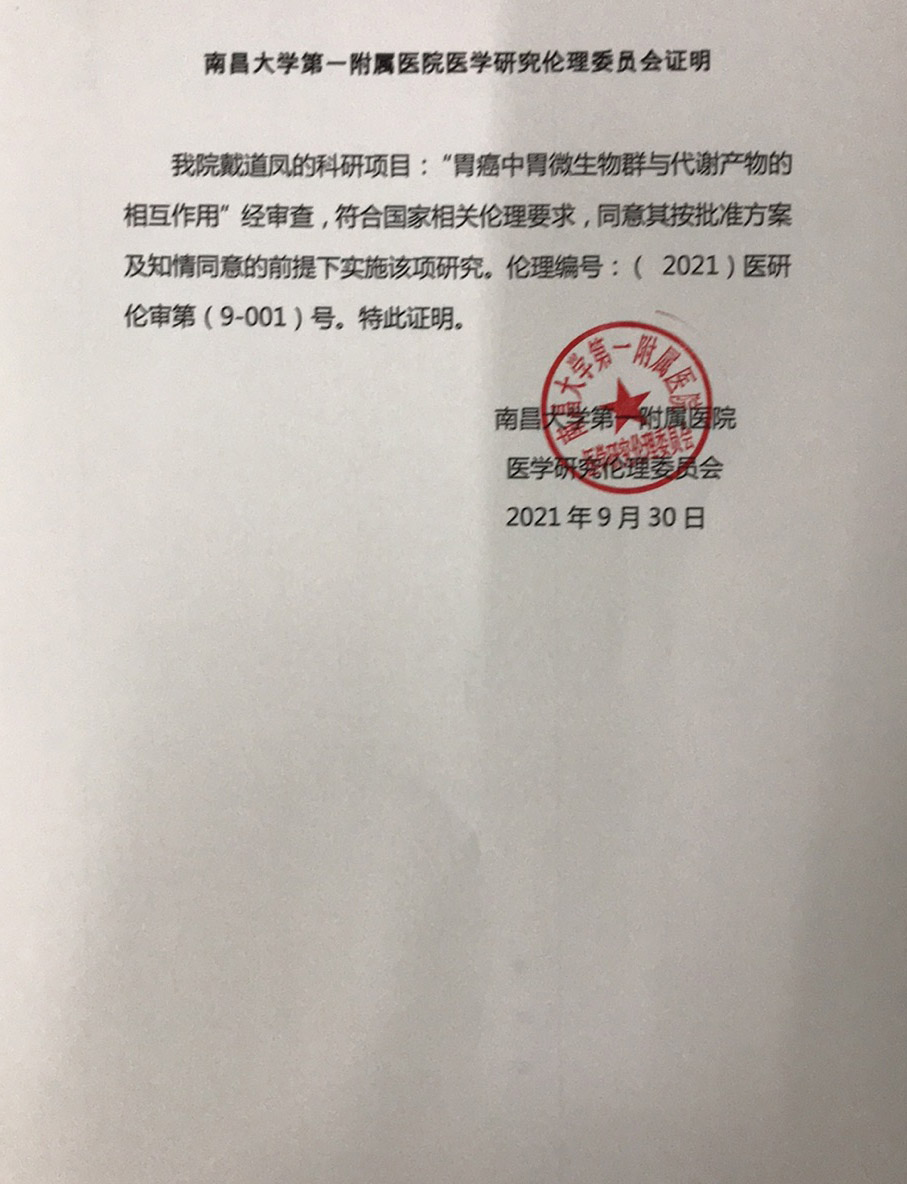

Supplement: Supplementary file 12 — Ethics statement-2 [file 41419_2021_4396_MOESM12_ESM.jpg]
