# Supplementary material for: Interactions between gastric microbiota and metabolites in gastric cancer
Source: Cell Death Dis. 2021 Nov 24;12(12):1104. doi: 10.1038/s41419-021-04396-y (PMC8613192; doi:10.1038/s41419-021-04396-y)
Supplement: Supplementary file 9 — cddis-author-contribution-form [file 41419_2021_4396_MOESM9_ESM.pdf]

DECLARATION OF CONTRIBUTIONS TO ARTICLE **ADMC**

Manuscript Number: CDDIS-21-1862R

Journal Name: *Cell Death & Disease* (the 'Journal')

Proposed Title of the Contribution: Interactions between gastric microbiota and metabolites in gastric cancer (the 'Contribution)

Author(s): Daofeng Dai, Yan Yang, Jieqing Yu, Tianfeng Dang, Wenjing Qin, Lisong Teng, Jing Ye, Hongqun Jiang (the 'Authors')

For all *CDDis* articles, each person named as an author in the published version must be able to show he or she has contributed substantially to the article.

Authorship credit should be based on 1) substantial contributions to conception and design, acquisition of data, or analysis and interpretation of data; 2) drafting the article or revising it critically for important intellectual content; and 3) final approval of the version to be published. Authors should meet conditions 1, 2 and 3.

Any person who cannot be shown to have made a substantial contribution to the article cannot be listed as an author in the final version. The name of any person who is deemed to have made a minor contribution can, however, appear in the Acknowledgments section of the article.

Please complete the table below to indicate the contributions of all named authors to the manuscript.

Author Full Name:	Specification of Contribution to the Manuscript:
Daofeng Dai	designed the study, performed the experiments, performed analysis and interpretation, drafted the manuscript, manuscript revision, approved the submitted version
Yan Yang	designed the study, collected samples, performed the experiments, manuscript revision, approved the submitted version
Jieqing Yu	collected samples, manuscript revision, approved the submitted version
Tianfeng Dang	performed the experiments, manuscript revision, approved the submitted version
Wenjing Qin	collected samples, manuscript revision, approved the submitted version
Lisong Teng	designed the study, collected samples, performed the experiments, supervised the study, manuscript revision, approved the submitted version
Jing Ye	designed the study, supervised the study, manuscript revision, approved the submitted version
Hongqun Jiang	designed the study, supervised the study, manuscript revision, approved the submitted version

Please complete the table below to indicate the contributions of all named authors to the figures.

Figure 1:

Daofeng Dai, Yan Yang, Jieqing Yu, Tianfeng Dang, Lisong Teng, Jing Ye, Hongqun Jiang

Figure 2:

Daofeng Dai, Yan Yang, Jieqing Yu, Tianfeng Dang, Lisong Teng, Jing Ye, Hongqun Jiang

Figure 3:

Daofeng Dai, Yan Yang, Jieqing Yu, Tianfeng Dang, Lisong Teng, Jing Ye, Hongqun Jiang

Figure 4:

Daofeng Dai, Yan Yang, Jieqing Yu, Tianfeng Dang, Lisong Teng, Jing Ye, Hongqun Jiang

Figure 5:

Daofeng Dai, Yan Yang, Jieqing Yu, Tianfeng Dang, Wenjing Qin, Lisong Teng, Jing Ye, Hongqun Jiang

Figure 6:

Daofeng Dai, Yan Yang, Jieqing Yu, Tianfeng Dang, Lisong Teng, Jing Ye, Hongqun Jiang

Signed for and on behalf of the Author(s):	Print Name:	Date:
Hongqun Jiang	Hongqun Jiang	2021. 11.02
