# Supplementary material for: Interactions between gastric microbiota and metabolites in gastric cancer
Source: Cell Death Dis. 2021 Nov 24;12(12):1104. doi: 10.1038/s41419-021-04396-y (PMC8613192; doi:10.1038/s41419-021-04396-y)
Supplement: Supplementary file 10 — Agreement_Author_Change [file 41419_2021_4396_MOESM10_ESM.pdf]

Addition of an author for the manuscript (CDDIS-21-1862R)

2 封[illegible]

发件人: yangyan1222

收件人: 戴道凤

2021年11月2日 上午 11:55

Dear Dr Dai,

I agree to add Dr Wenjing Qin as the co-author of the article.

-----原始邮件-----
发件人:"戴道凤" <ddf2010@alumni.sjtu.edu.cn>
发送时间:2021-11-02 09:35:55 (星期二)
收件人: yangyan1222@zju.edu.cn
抄送:
主题: Addition of an author for the manuscript (CDDIS-21-1862R)

Dear Dr Yang,

Dr Wenjing Qin made some contributions to the collection of gastric cancer tissues which were used for validation of the two differential metabolites, namely 1-methylnicotinamide and N-acetyl-D-glucosamine-6-phosphate. She hope that her name can be added to the author list of the manuscript (CDDIS-21-1862R). As the co-author of the manuscript, your opinion will be respected. I am waiting for your opinion.

Sincerely,

Daofeng Dai

隐藏引用文本 - 回复 - 全部回复 - 转发 - 更多操作选项

戴道凤 2021年11月2日 上午 9:35

Addition of an author for the manuscript (CDDIS-21-1862R)

3 封邮

发件人: yu jiezi

收件人: ddf2010

2021年11月2日 下午 2:36

Agree

发自 网易邮箱大师

---- 回复的原邮件 ----

发件人	戴道风<ddf2010@alumni.sjtu.edu.cn>
日期	2021年11月02日 09:37
收件人	yu jiezi<yu_jiezi@126.com>
主题	Addition of an author for the manuscript (CDDIS-21-1862R)

Dear Jieqing,

Dr Wenjing Qin made some contributions to the collection of gastric cancer tissues which were used for validation of the two differential metabolites, namely 1-methylnicotinamide and N-acetyl-D-glucosamine-6-phosphate. She hope that her name can be added to the author list of the manuscript (CDDIS-21-1862R). As the co-author of the manuscript, your opinion will be respected. I am waiting for your opinion.

Sincerely,

Daofeng Dai

Addition of an author for the manuscript (CDDIS-21-1862R)

2 封邮

发件人: audrey0313

收件人: 戴道凤

2021年11月2日 上午 9:58

Agree!

---- Replied Message ----

From	戴道凤<ddf2010@alumni.sjtu.edu.cn>
Date	11/02/2021 09:36
To	audrey0313<audrey0313@163.com>
Subject	Addition of an author for the manuscript (CDDIS-21-1862R)

Dear Tianfeng,

Dr Wenjing Qin made some contributions to the collection of gastric cancer tissues which were used for validation of the two differential metabolites, namely 1-methylnicotinamide and N-acetyl-D-glucosamine-6-phosphate. She hope that her name can be added to the author list of the manuscript (CDDIS-21-1862R). As the co-author of the manuscript, your opinion will be respected. I am waiting for your opinion.

Sincerely,

Daofeng Dai

Addition of an author for the manuscript (CDDIS-21-1862R)

2 封邮

发件人: lsteng

收件人: 戴道凤

2021年11月2日 上午 11:51

Dear Dr Dai,

I agree to add Dr Wenjing Qin as co-author of the article.

Sincerely,

> -----原始邮件-----
> **发件人:**"戴道凤" <ddf2010@alumni.sjtu.edu.cn>
> **发送时间:**2021-11-02 09:38:37 (星期二)
> **收件人:** lsteng@zju.edu.cn
> **抄送:**
> **主题:** Addition of an author for the manuscript (CDDIS-21-1862R)
>
> Dear Prof. Teng,
>
> Dr Wenjing Qin made some contributions to the collection of gastric cancer tissues which were used for validation of the two differential metabolites, namely 1-methylnicotinamide and N-acetyl-D-glucosamine-6-phosphate. She hope that her name can be added to the author list of the manuscript (CDDIS-21-1862R). As the co-author of the manuscript, your opinion will be respected. I am waiting for your opinion.
>
> Sincerely,
>
> Daofeng Dai

隐藏引用文本 - 回复 - 全部回复 - 转发 - 更多操作选项

戴道凤 2021年11月2日 上午 9:38

Addition of an author for the manuscript (CDDIS-21-1862R)

发件人: yjholly

收件人: 戴道凤

2021年11月2日 上午 9

agree

Jing Ye

发自我的iPhone

------------------ Original ------------------

From: 戴道凤 <ddf2010@alumni.sjtu.edu.cn>
Date: Tue,Nov 2,2021 9:44 AM
To: yjholly <yjholly@email.ncu.edu.cn>
Subject: Re: Addition of an author for the manuscript (CDDIS-21-1862R)

Dear Prof. Ye,

Dr Wenjing Qin made some contributions to the collection of gastric cancer tissues which were used for validation of the two differential metabolites, namely 1-methylnicotinamide and N-acetyl-D-glucosamine-6-phosphate. She hope that her name can be added to the author list o the manuscript (CDDIS-21-1862R). As the co-author of the manuscript, your opinion will be respected. I am waiting for your opinion.

Sincerely,

Daofeng Dai

Addition of an author for the manuscript (CDDIS-21-1862R)

2 封邮

发件人: jianghq

收件人: 戴道凤

2021年11月2日 上午 10:31

Agree

------------------ Original ------------------

From: "戴道凤"<ddf2010@alumni.sjtu.edu.cn>;
Date: Tue, Nov 2, 2021 09:45 AM
To: "jianghq"<jianghq@ncu.edu.cn>;
Subject: Addition of an author for the manuscript (CDDIS-21-1862R)

Dear Prof. Jiang,

Dr Wenjing Qin made some contributions to the collection of gastric cancer tissues which were used for validation of the two differential metabolites, namely 1-methylnicotinamide and N-acetyl-D-glucosamine-6-phosphate. She hope that her name can be added to the author list of the manuscript (CDDIS-21-1862R). As the co-author of the manuscript, your opinion will be respected. I am waiting for your opinion.

Sincerely,

Daofeng Dai
